# Budding yeast Rap1, but not telomeric DNA, is inhibitory for multiple stages of DNA replication in vitro

**DOI:** 10.1093/nar/gkab416

**Published:** 2021-05-28

**Authors:** Max E Douglas, John F X Diffley

**Affiliations:** Telomere Biology Laboratory, The Institute of Cancer Research, 237 Fulham Road, London SW3 6JB, UK; Chromosome Replication Laboratory, The Francis Crick Institute, 1 Midland Road, London, NW1 1AT, UK

## Abstract

Telomeres are copied and reassembled each cell division cycle through a multistep process called telomere replication. Most telomeric DNA is duplicated semiconservatively during this process, but replication forks frequently pause or stall at telomeres in yeast, mouse and human cells, potentially causing chronic telomere shortening or loss in a single cell cycle. We have investigated the cause of this effect by examining the replication of telomeric templates in vitro. Using a reconstituted assay for eukaryotic DNA replication in which a complete eukaryotic replisome is assembled and activated with purified proteins, we show that budding yeast telomeric DNA is efficiently duplicated in vitro unless the telomere binding protein Rap1 is present. Rap1 acts as a roadblock that prevents replisome progression and leading strand synthesis, but also potently inhibits lagging strand telomere replication behind the fork. Both defects can be mitigated by the Pif1 helicase. Our results suggest that GC-rich sequences do not inhibit DNA replication per se, and that in the absence of accessory factors, telomere binding proteins can inhibit multiple, distinct steps in the replication process.

## INTRODUCTION

Telomeres are dynamic nucleoprotein structures that protect and maintain the ends of eukaryotic chromosomes and are composed of short GC-rich sequences repeated over hundreds (yeasts) or thousands (humans) of base pairs. Telomeric defects or deletions from even a single chromosome end are associated with loss of terminal DNA sequences, breakage-fusion-bridge cycles and tetraploidisation, driving karyotype changes and genome instability (1,2). To prevent such deleterious outcomes, telomere replication is required to faithfully maintain these structures from one cellular generation to the next; however, in yeasts, mouse and human cells replication forks frequently pause or stall during this process (3–5). At most loci, loss of genetic material after replication fork stalling is prevented by a second fork approaching from the opposite direction. However, telomeres are terminally positioned and replicated by a single replication fork in most instances (5) leaving them particularly vulnerable to stalling events and associated DNA loss. Accessory proteins including Pif- and RecQ-family helicases have been shown to play important roles in preventing telomeric replication stress (3,6,7) but the molecular basis of how replication is inhibited or blocked at the chromosome end remains poorly understood.

A diverse range of telomeric properties have been proposed to contribute to this effect, including t-loops (8) telomeric compaction (9), nuclear envelop attachment (9), protein and RNA factors bound to telomeric DNA (10–12) and the inherent properties of telomeric sequences (13). Whilst it is likely that the overall impact of telomeres on the replication fork results from a combination of these properties, understanding their relative contribution, and which stages of the replication process are affected is important to gain a comprehensive understanding of how telomeres are replicated successfully.

Much work in this area has focussed on the impact of telomeric DNA itself. Single stranded G-rich sequences from yeast and human telomeres can assemble into stacks of four planar guanosine residues called G-quadruplexes (G4s) (14). G4s can in turn block DNA polymerases in vitro (15), suggesting that the inherent properties of telomeric templates may inhibit DNA replication directly. Consistent with this idea, G4-binding molecules recognise budding yeast and human telomeres during S-phase (16,17), and mouse or human cells treated with G4-stabilising compounds or defective in G4-specific helicases show telomere fragility or loss (5,6,8). Notably, as genetic approaches can only address the impact of telomeric DNA indirectly due to the wide array of DNA binding factors in the cell, and in vitro analyses have so far been limited to simple polymerase assays (18), whether replication is directly inhibited by telomeric sequences is currently unclear.

A second potential barrier likely to be encountered at most telomeric positions is the collection of DNA-bound proteins that coordinate essentially all aspects of telomere function. Elsewhere in the genome, DNA-bound proteins act as replication roadblocks that stall or pause the replisome (19). Genetic and biochemical evidence suggest budding yeast Rap1 and the mammalian shelterin complex may act in a similar manner (11,12,20); however, Rap1 is essential, and shelterin facilitates DNA replication in vivo by recruiting BLM to the chromosome end (5), complicating genetic experiments aiming to characterise this effect. Our mechanistic understanding of which aspects of DNA replication are affected by telomere-bound proteins and how blocks or barriers are overcome is therefore limited.

To examine directly the impact of different telomeric properties on DNA replication, we have analysed budding yeast telomere replication in vitro. Using a replication system in which a complete eukaryotic replisome is reconstituted with purified proteins, we show that both leading and lagging strand synthesis proceed to completion across budding yeast telomeric DNA unless the telomere binding protein Rap1 is present. Rap1 acts as a replication roadblock that inhibits fork progression, but also induces a penetrant defect in lagging strand synthesis behind the fork. Our results suggest that unwinding of budding yeast telomeric sequences by the replisome alone is unlikely to inhibit DNA replication, but that DNA-bound proteins can act as potent inhibitors of multiple steps in the telomere replication process.

## MATERIALS AND METHODS

### DNA construct assembly

*In vitro* replication of control templates lacking telomeric DNA employed construct MD154, which is based on a GC209, a pUC19 vector containing an insert composed of two inverted optimal ORC binding sites spaced 70 bp apart (21). To construct MD154, high scoring matches to the ARS consensus sequence (ACS) outside of this insert were mutated, and fragments of selected yeast open reading frames lacking high scoring ACS matches were added, increasing the construct size to 8165 bp. Reactions containing budding yeast telomeric DNA employed construct MD155, which is identical to MD154 except for the inclusion of a 379 bp DNA fragment from vector YLP108CA-4 (22) between NotI and PacI restriction sites, which includes 325 bp of budding yeast telomeric DNA. The sequences of MD154 and MD155 are provided in the ‘Supplementary information’ file. To assemble a construct for expression of full length Rap1, Rap1 was amplified from budding yeast cells directly using oligonucleotides oMD181 (tagtagGAATTCATGTCTAGTCCAGATGATTTTGAAACTGC) and oMD182 (tagtagCTCGAGTCATAACAGGTCCTTCTCAAAAAATCTTTTCC), and the resulting PCR product was digested with XhoI and EcoRI (NEB) and cloned into the pGEX-6P-1 vector digested with the same enzymes to make plasmid pTBL044.

### Preparation of DNA templates for in vitro replication

**Figure 1.**
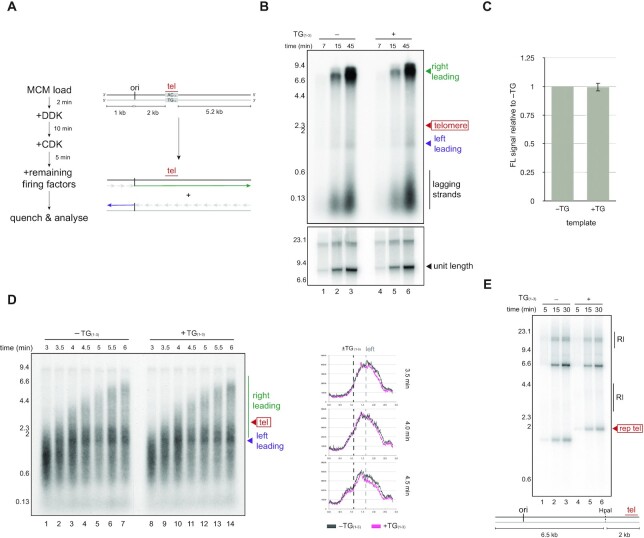
Leading strand synthesis and replisome progression are not inhibited by budding yeast telomeric DNA. (**A**) Outline of replication reactions, and schematic showing the templates used and expected leading and lagging strand products. (**B**) Replication reactions performed with the templates indicated for the times indicated were analysed by denaturing alkaline agarose electrophoresis (top panel) and native agarose electrophoresis (bottom panel). (**C**) quantification showing intensity of unit length products imaged after native agarose electrophoresis, formed in the presence or absence of telomeric DNA after 15 min of replication. Average of three independent experiments. Error bars show standard deviation. (**D**) Alkaline agarose electrophoresis analysis of replication products from a pulse chase analysis in the absence of polymerase δ with the templates indicated. Chase was added 2.5 min after the addition of firing factors, and samples were then taken at the time points indicated. Time indicates minutes after firing factor addition. Lane profiles show the position of the left leading strand (‘left’) and telomeric DNA (±TG1–3). (**E**) Replication reactions were performed with the template depicted in the presence or absence of telomeric DNA. Reaction products were purified, digested with HpaI and analysed by native agarose electrophoresis. Expected size of replication intermediates (RIs), reflecting forked DNA structures is indicated.

To linearise MD154 and MD155 prior to replication, 10–15 μg of plasmid vector was digested in a final volume of 40 μl with 80 units of restriction enzyme (NEB) for 3 h at 37°C. ScaI-HF digested DNA was used for all reactions except those in Figure 1D, where PacI was used. Reactions were quenched with an equal volume of ‘stop mix’, containing 0.1% SDS (Sigma), 0.2 mg/ml Proteinase K in Tris–HCl pH 7.5 (Sigma) and 40 mM EDTA. After incubation at 37°C for 20 min, an equal volume of phenol:chloroform:isoamyl alcohol 25:24:1 saturated with 10 mM Tris, pH 8.0, 1 mM EDTA (Sigma) was added and the aqueous phase collected after centrifugation for 5 min. The solution was supplemented with NaCl to 200 mM and two volumes of −20°C ethanol added before centrifugation at 4°C for 20 min. Ethanol precipitates were washed twice with room temperature 70% (v/v) ethanol, air dried and resuspended in 10–15 μl 1× Tris–EDTA (TE) solution and stored at −20°C before use.

### Protein expression and purification

ORC, Cdc6, Mcm2-7:Cdt1, DDK, Dpb11, GINS, Cdc45, polymerase ϵ, CDK, Ctf4, RPA, TopoI, PCNA, Mcm10, polymerase δ, polymerase α/primase, Sld3/Sld7, Sld2, Csm3/Tof1, Mrc1 and RFC were expressed and purified as described (23–25). Fen1, Cdc9 and Pif1 were expressed and purified as described (26,27). To purify full length Rap1, BL21-CodonPlus (DE3)-RIL E. coli cells (Agilent technologies) transformed with plasmid pTBL044 were grown in LB medium at 25°C to a density corresponding to OD600 0.6–0.8. Isopropyl-ß-d-thio-galactopyranoside (IPTG) was added to 1 mM. After 4 h at 25°C, cells were collected by centrifugation, washed once with phosphate buffered saline (PBS), frozen in liquid nitrogen and stored at −80°C. For lysis, a cell pellet from a starting culture volume of 2 l was resuspended in 30 ml lysis buffer (50 mM Tris 7.4, 0.02% NP-40-substitute, 1 mM EDTA, 10% glycerol and 1 mM DTT) + 500 mM NaCl, supplemented with 0.5 mM AEBSF, 10 μg/ml Leupeptin, 10 μg/ml Pepstatin A. Lysozyme (Sigma) was added to 0.5 mg/ml and the sample incubated on ice for 20 min. After sonication for 5 min (2 s on, 3 s off), crude lysate was centrifuged at 23 600 g for 30 min, 4°C, and the supernatant mixed with 1 ml packed volume of glutathione sepharose (Sigma) prewashed in lysis buffer + 500 mM NaCl. After 1.5 h incubation at 4°C, beads were washed with ∼50 column volumes (CV) lysis buffer + 500 mM NaCl, resuspended in 1 CV lysis buffer + 500 mM NaCl supplemented with 0.04 mg/ml 3C protease and incubated at room temperature for 30 min. The flow through was collected, and additional cleaved protein was eluted with four consecutive CVs of lysis buffer + 500 mM NaCl. Rap1-containing eluates were pooled, slowly diluted 3.3× with lysis buffer lacking NaCl to a final salt concentration of 150 mM. and applied to a Mono Q 5/50 GL column (Sigma) preequilibrated with lysis buffer + 150 mM NaCl. Bound protein was eluted over a 20 CV gradient from 150 to 1000 mM NaCl, Rap1 containing fractions were pooled, concentrated to 0.5 ml on a 30 kDa cutoff ultra-4 centrifugal filter (Amicon) and loaded onto a Superdex 200 Increase 10/300 GL column (Sigma) preequilibrated with lysis buffer +150 mM NaCl. Rap1 containing fractions were pooled, concentrated, frozen in liquid nitrogen and stored at −80°C prior to use.

### *In vitro* replication reactions

MCM loading was carried out in reactions containing a final concentration of 25 mM HEPES pH 7.6, 10 mM MgOAc, 0.02% (v/v) NP-40-substitute, 5% glycerol, 100 mM K-glutamate, 1 mM DTT, 5 mM ATP, 5 nM DNA template, 20 nM ORC, 50 nM Cdc6 and 100 nM Mcm2-7:Cdt1, and incubated for 2 min at 24°C prior to the addition of DDK to 80 nM. After 10 further minutes at 24°C, CDK was added to 20 nM. After 5 further minutes at 24°C, replication buffer was added, the temperature increased to 30°C, and a mix of firing and replication factors added. The final concentrations of reaction components was as follows: 25 mM HEPES pH 7.6, 10 mM MgOAc, 0.02% (v/v) NP-40-substitute, 2.5% glycerol, 220 mM K-glutamate, 0.5 mM DTT, 4.5 mM ATP, 30 μM each dATP, dCTP, dGTP, dTTP, 400 μM each UTP, GTP, CTP, 33 nM α-^32^P- labelled dCTP, 2.5 nM DNA template, 10 nM ORC, 25 nM Cdc6, 50 nM Mcm2–7:Cdt1, 40 nM DDK, 45 nM Dpb11, 20 nM GINS, 40 nM Cdc45, 30 nM polymerase ϵ, 30 nM CDK, 20 nM Ctf4, 50 nM RPA, 10 nM TopoI, 70 nM PCNA, 2.5 nM Mcm10, 4 nM polymerase δ, 40 nM polymerase α/primase, 25 nM Sld3/Sld7, 40 nM Sld2, 40 nM Csm3/Tof1, 10 nM Mrc1, 20 nM RFC.

### Modified *in vitro* replication reactions

**Figure 2.**
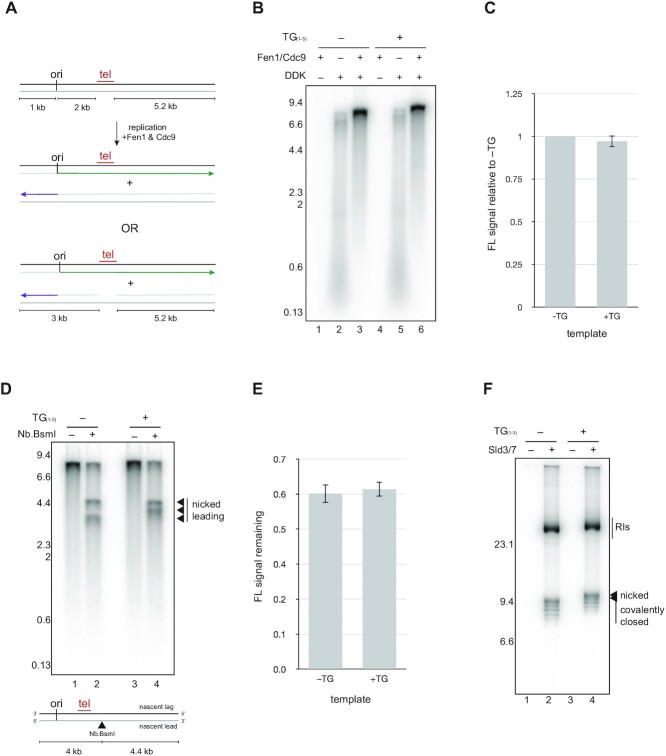
Lagging strand replication proceeds to completion in the presence of budding yeast telomeric DNA. (**A**) Schematic of templates and expected products with or without defects in lagging strand replication over telomeric DNA. (**B**) Replication reactions were performed for 1 h in the presence or absence of Fen1/Cdc9 and DDK as indicated and analysed by alkaline agarose electrophoresis. (**C**) Quantification of unit length products in the presence or absence of telomeric DNA. Average of three independent experiments. Error bars show standard deviation. (**D**) Replication products from reactions containing Fen1/Cdc9 and the templates indicated were treated with the nicking enzyme Nb.BsmI as indicated and analysed by denaturing alkaline electrophoresis. Replication was performed for 1 h. Diagram shows the position of the Nb.BsmI site relative to other template features. (**E**) Quantification showing the fraction of unit length products remaining after Nb.BsmI treatment of replication products from telomeric or non-telomeric templates. Average of three independent experiments. Error bars show the standard deviation. (**F**) Replication reactions were carried out for 1 h with the circular templates indicated in the presence or absence of the firing factor complex Sld3/7. Products were analysed by native agarose electrophoresis. Reactions lacking Sld3/7 are stalled at an early stage of CMG assembly and in this context are essentially equivalent to reactions lacking DDK.

For replication reactions lacking polymerase δ, the final concentration of K-glutamate was reduced to 100 mM. For replication reactions containing Rap1, Rap1 was added after replication buffer and the reaction incubated for 5 min at 30°C prior to firing factor addition. For the pulse chase reaction in Figure 1D, the concentration of dCTP was reduced to 5 μM, and increased to 250 μM after 2.5 min. For the reactions in Figure 2B, Figure 2D, Figure 4 and Figures 5F and G, 60 nM Fen1 and 60 nM Cdc9 were added in addition to the mix of replication proteins listed above. For reactions containing Pif1, Pif1 was added at the concentration indicated, with the mix of replication proteins listed above. For the replication reactions in Figure 2F, 2.5 nM circular DNA template was used and 60 nM Fen1, 60 nM Cdc9 and 10 nM TopoII were added in addition to the mix of replication proteins listed above.

### Molecular weight markers

12.5 μg HindIII-digested phage lambda DNA was dephoshorylated in 1x Cutsmart buffer (NEB) with 10 U Quick-CIP phosphatase (NEB) in a final volume of 30 μl for 1 h at 37°C. After heat inactivation at 80°C for 2 mins, DNA was purified through a spin column (Roche) and 1.4 μg was resuspended in 1x PNK buffer (NEB) supplemented with 1.35 mM γ-^32^P- labelled ATP and 20 U polynucleotide kinase (NEB). After incubation at 37°C for 1 h and heat inactivation at 65°C for 20 min, the sample was desalted over a G-50 microspin column (Sigma) and stored at −20°C prior to use.

### Processing and analysis of replication products

Replication reactions were quenched by adding an equal volume of stop buffer (see ‘Preparation of DNA templates for in vitro replication’) and incubated at 37°C for 15–20 min. An equal volume of Phenol:Chloroform:Isoamyl Alcohol 25:24:1 saturated with 10 mM Tris, pH 8.0, 1 mM EDTA was added, the aqueous phase was collected after centrifugation for 5 min and the sample desalted using a G-50 microspin column before being processed as follows: samples for analysis by denaturing alkaline agarose electrophoresis were supplemented with 20 mM EDTA, 0.5% sucrose and 50 mM NaOH, incubated at room temperature for 10 mins and loaded onto a 0.7% agarose gel supplemented with 2 mM EDTA and 30 mM NaOH, which was run at 0.75 V/cm for 16 h in 2 mM EDTA and 30 mM NaOH. Gels were fixed in 5% trichloroacetic acid for 30–60 min, dried onto filter paper and imaged by phosphorimaging and autoradiography. Samples for Figure 1E were diluted into 1× cutsmart buffer (NEB) and 2.5 U of HpaI added per 5 μl replication products. After incubation at 37°C for 30 mins, EDTA was added to 20 mM and loading dye (NEB) added to 1×. The sample was run on a 1% agarose TAE gel at 0.75 V/cm for 18 h, which was then dried and imaged by phosphorimaging. Samples for Figures 2D, 4C and 5G were resuspended in 1× buffer 3.1 (NEB) and 5 U Nb.BsmI enzyme added per 5 μl replication products. After incubation at 65°C for 30 min, EDTA was added to 20 mM, the sample supplemented with 0.5% sucrose and 50 mM NaOH, and analysed by denaturing alkaline electrophoresis as above. Samples for Figure 2F were supplemented with NaCl to 200 mM, precipitated by the addition of two volumes of ice cold ethanol, centrifuged for 20 min at 4°C, washed 2× with room temperature 70% ethanol and the pellet resuspended in 18 μl TE buffer and 2 μl topoisomerase IV buffer (Topogen) and 0.5 μl *Escherichia coli* Topoisomerase IV (Topogen) added. After incubation at 37°C for 20 min, loading dye (NEB) was added to 1x, and samples loaded onto a 0.6% agarose 1× TAE gel, which was run at 0.75 V/cm for 18 h, and then dried and imaged by phosphorimaging. For the 2D gel analysis in Figure 4E, replication products were run on a native agarose electrophoresis gel as above, the lane excised, soaked in 1 mM EDTA, 30 mM NaOH solution for 1–2 h and set in a 0.7% agarose gel supplemented with 2 mM EDTA and 30 mM NaOH, which was then run and analysed as above.

### Quantification of data

Gel densitometry was performed using original .gel files and ImageJ.

### Electrophoretic mobility shift assays

20 μg plasmid MD155 was digested with BamHI for 2 h at 37°C in a final volume of 100 μl, 1 μl quickCIP (NEB) phosphatase added and after a further 30 min the reaction quenched with 25 mM EDTA, 0.2% SDS and 0.2 mg/ml proteinase K. After 30 min, an equal volume of phenol:chloroform:isoamyl alcohol was added, the sample centrifuged at full speed for 5 min in a benchtop centrifuge and the supernatant supplemented with NaCl to a final concentration of 300 mM prior to ethanol precipitation as above. Precipitated DNA was resuspended in 10–20 μl 1× TE and run on an 8% tris–borate–EDTA (TBE) polyacrylamide gel, which was then stained with sybrsafe dye (Invitrogen). The band corresponding to the telomeric fragment was excised, fragmented and incubated at room temperature with 300 μl 1× TE supplemented with 300 mM NaCl overnight with shaking, prior to ethanol precipitation as above. Precipitated DNA was resuspended in 1× TE to a final concentration of 150 nM, and end labelled using polynucleotide kinase (PNK) for 1 h at 37°C in a reaction containing 1x PNK buffer (NEB), 40 nM DNA, 10 U PNK, 7.5 μM γ-^32^P-labelled ATP. PNK was inactivated by heating at 65°C for 2 min and the sample desalted over a G50 spin column (GE healthcare). For the EMSA, labelled DNA was incubated at a final concentration of 2.5 nM with the Rap1 and Pif1 concentrations indicated for 20 min in reactions containing 25 mM HEPES pH 7.6, 10 mM MgOAc, 0.02% (v/v) NP-40-substitute, 2.5% glycerol, 220 mM K-glutamate, 0.5 mM DTT, 4.5 mM ATP. Samples were supplemented with 0.5% sucrose and run on a 1.5% agarose 0.5× TBE gel, which was then dried and imaged by phosphorimaging.

## RESULTS

### Telomeric DNA does not inhibit replication fork progression or leading strand synthesis in vitro

To examine the causes of telomeric replication stress, we set out to study the replication of telomeric templates using a reconstituted assay for eukaryotic DNA replication (25). In this assay, a complete eukaryotic replisome is assembled from purified budding yeast proteins on templates that are initially double stranded and are melted and unwound in a stepwise manner (28). To unambiguously identify left and right replication forks, we employed reaction conditions that bias replication initiation towards origin sequences (29), and linear DNA templates containing an origin-like sequence (21) displaced to one end (Figure 1A). Denaturing alkaline agarose electrophoresis of products from reactions containing radiolabelled dCTP showed a dominant species of ∼7 kb and a less intense product at 1–1.5 kb after the addition of origin firing factors (hereafter ‘firing factors’), consistent with bidirectional leading strand replication proceeding from the origin to each template end (Figure 1B, lanes 1–3 and see diagram in Figure 1A). Lagging strand maturation factors Fen1 and Cdc9 were excluded in this particular experiment, leaving lagging strand products unligated at ∼100–500 bp in length.

To analyse the effect of telomeric DNA on the replication process, a complete 325 bp budding yeast telomere sequence (22) (Supplementary Figure S1) was inserted into the template at an internal site ∼2 kb to one side of the origin. As shown in Figure 1A, telomeric DNA in this position is copied by the rightward-moving replication fork and is oriented such that leading and lagging strands are templated by C- or G-rich telomeric DNA respectively, as *in vivo*. To determine whether replication was impaired by this sequence, synthesis of radiolabelled nascent strands was followed via denaturing and native agarose electrophoresis. As shown in Figure 1B and C, telomeric DNA had no effect on either the profile of products after alkaline electrophoresis, or the amount of fully replicated products compared with a control template lacking an insert (compare lanes 1–3 and 4–6), suggesting leading strand synthesis and replisome progression proceed apparently unhindered across budding yeast telomeric DNA in vitro. To confirm that this was the case, we analysed the progression of a subset of replication forks by initiating replication with a high proportion of radiolabelled dCTP, before adding a chase of unlabelled dCTP less than three minutes later. In good agreement with Figure 1B and C, this pulse-chase analysis shows that telomeric DNA had no appreciable effect on the profile or distribution of nascent leading strands as they progressed through the insertion site (Figure 1D, compare profile of grey and pink lines).

Telomeric DNA was positioned internally in these experiments. To exclude that this was responsible for the efficient replication we observed, starting plasmids were digested such that the telomeric tract was positioned proximal to the template end (see diagram in Figure 1E). Telomeric DNA was also copied efficiently in this position, with no increase in replication intermediates (which reflect forked DNA structures) accumulated on a terminal fragment of the template after native agarose electrophoresis (Figure 1E, compare terminal fragment, and replication intermediates—‘RI’—in lanes 1–3 and 4–6). We conclude that replisome progression and leading strand synthesis proceed efficiently across budding yeast telomeric DNA positioned at an internal or end-proximal position in vitro.

### Lagging strand replication proceeds to completion across budding yeast telomeric DNA *in vitro*

In the absence of maturation factors, lagging strand products from across the template remain unligated as individual Okazaki fragments (OFs) that are indistinguishable from one-another in the experimental approach used above. To examine the lagging strand in our reactions, we therefore added Fen1 and Cdc9, enabling processing and ligation of OFs together with the leading strand of the opposing replication fork (26,27) (Figure 2A). In control reactions containing these factors, we observed a new replication-dependent species corresponding to the size of a fully replicated and ligated 8.2 kb linear product, formed at the expense of individual OFs (Figure 2B, compare lanes 2 and 3). If OF synthesis were defective over the telomere, the resultant gap on the nascent lagging strand would give rise to two additional products approximately 5 and 3 kb in length (see diagram in Figure 2A). However, Figure 2B and C shows that the profile of products was largely unchanged and the amount fully replicated was not significantly different when a telomeric template was used. To exclude that a defect in lagging strand synthesis in these assays is obscured by nascent leading strands (which also run as unit length), purified replication products were treated with a site-specific nickase, Nb.BsmI, which targets a single site ∼600 bp downstream of telomeric DNA on the nascent leading strand (see diagram in Figure 2D). Consistent with Figure 2B and C, the fraction of unit length products resistant to Nb.BsmI was not affected by the presence of telomeric DNA (Figure 2D and E). Although we cannot rule out transient defects with these assays, the data collectively show that lagging strand synthesis across budding yeast telomeric DNA proceeds to completion in the majority of instances in vitro. Consistent with this conclusion, native agarose electrophoresis of replication products from reactions containing circular templates shows radiolabelled covalently closed topoisomers in the presence or absence of telomeric DNA, which can only form if both leading and lagging strands have been synthesised completely (Figure 2F).

### Phen-DC3 is inhibitory for lagging strand telomere replication

Whilst we did not reproducibly detect lagging strand defects in the analysis above, lane scans occasionally detected a faint shoulder at the position of the telomere, suggesting lagging strand defects may occur in vitro but are infrequent (see e.g. Supplementary Figure S2a; 2/6 experiments examined). Prompted by previous work showing that budding yeast telomeric sequences can form G4s (14), we examined the effect of adding the G4-stabilising agent Phen-DC3 to our reactions. Replication was generally inhibited by Phen-DC3 concentrations >60 nM (Supplementary Figure S2b). However, whilst 60 nM Phen-DC3 had only a minor effect in reactions containing a control template, we observed two additional bands of approximately 5 and 3 kb when a telomeric template was used, suggesting lagging strand replication over the telomere was defective (Supplementary Figure S2c, arrow heads. compare lanes 1–2 and 4–5).

Our telomeric insert contains 325 bp of telomeric DNA and a short non-telomeric G-rich sequence originating from the DNA fragment used for cloning (Supplementary Figure S2c diagram). Both elements are likely to contribute to the replication defect above to some extent, as insertion of only the non-telomeric sequence into the control vector induced the appearance of 5 and 3 kb bands in the presence of Phen-DC3 that were weaker and narrower than when telomeric DNA was also present (Supplementary Figure S2c, compare lanes 5 and 6). Thus, G4-prone sequences can interfere with lagging strand replication when stabilised by Phen-DC3 and the presence of telomeric DNA enhances this effect.

### Rap1 is sufficient to block DNA replication in a sequence-specific manner.

Figures 1 and 2 show that the inherent properties of telomeric DNA alone are unlikely to cause the replication defects observed at budding yeast telomeres each cell cycle. We therefore turned our attention to telomere binding proteins, which have also been proposed to inhibit DNA replication (11,12,20). The primary protein component of budding yeast telomeres is the general regulatory factor Rap1, which recognises a 13-bp consensus motif via tandem Myb domains (30). Rap1 has previously been implicated in telomeric replication stress (12,20). However, genetic experiments aiming to characterise this effect are complex due to the role of Rap1 as a transcriptional regulator (31), and the impact of Rap1 on different stages of the replication process is currently unclear.

**Figure 3.**
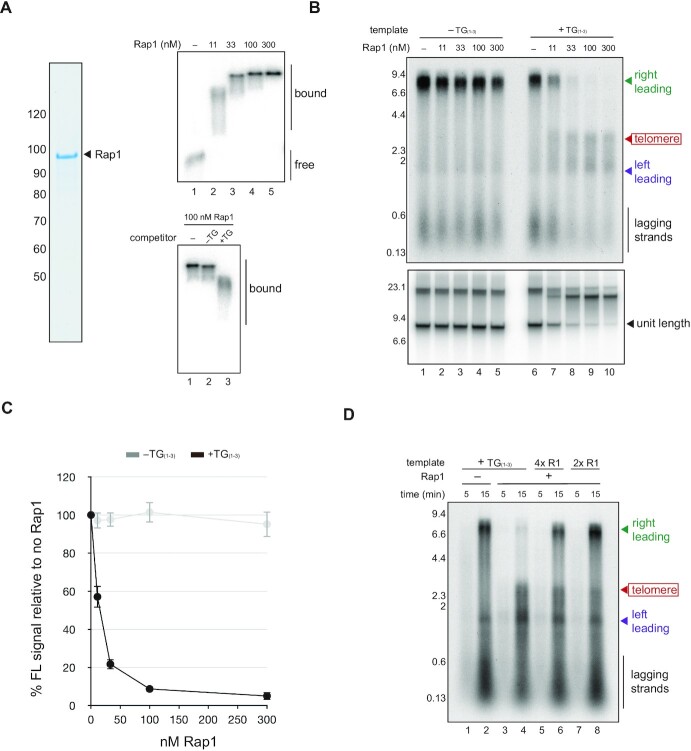
Rap1 acts as a replication roadblock on telomeric DNA. (**A**) A radiolabelled 361 bp fragment of telomeric DNA was incubated for 15 min with purified Rap1 as indicated under DNA and buffer conditions used in the replication assay and analysed by native agarose electrophoresis. Molecular weight markers on the Rap1 gel indicated in kilodaltons. Bottom panel shows reactions performed as for the top panel except a 5-fold molar excess of telomeric or non-telomeric DNA was added. (**B**) Replication reactions performed with telomeric or non-telomeric templates in the presence of the Rap1 concentrations indicated were analysed by alkaline electrophoresis (top panel) and native agarose electrophoresis (bottom panel). (**C**) Quantification of unit length products after native agarose electrophoresis of replication products formed with the templates and Rap1 concentrations indicated. Average of three independent experiments. Error bars show standard deviation. (**D**) Replication reactions performed in the presence or absence of 100 nM Rap1 and the templates indicated were analysed by alkaline agarose electrophoresis. Templates ‘4× R1’ and ‘2× R1’ contain either four or two consensus Rap1 binding sites in place of telomeric DNA (TG_(1–3)_), which contains seven consensus Rap1 binding sites.

The telomeric fragment examined above contains seven consensus Rap1 binding sites (Supplementary Figure S1) and was effectively bound by purified Rap1 under reaction conditions used in the replication assay (Figure 3A, top panel). The migration pattern observed via EMSA suggests each DNA fragment binds at least one Rap1 molecule at 11 nM, and that more sites are occupied as Rap1 concentration is increased (top panel, compare lanes 2–5). Binding was sensitive to an excess of telomeric competitor but largely unaffected by non-telomeric DNA, suggesting it is sequence specific, as expected (Figure 3A, bottom panel). To examine DNA replication under these conditions, *in vitro* replication was carried out for 15 min in the absence of Fen1 and Cdc9 and products analysed by native and denaturing agarose electrophoresis. As shown in Figure 3B, Rap1 caused rightward-moving leading strand to accumulate on telomeric DNA (top panel), and the appearance of a slowly migrating species after native electrophoresis (bottom panel) consistent with an accumulation of stalled or paused replication forks. Notably, the magnitude of this effect increased with Rap1 concentration (Figure 3B, compare lanes 7–10, Figure 3C), suggesting the number of Rap1 molecules bound to the template may determine replication efficiency in this system. To examine this idea, we generated constructs in which telomeric DNA was replaced with either four or two consensus Rap1 binding sites. As shown in Figure 3D, decreasing the number of binding sites increased the number of forks able to bypass the insert and reach the template end (Figure 3D, compare lanes 2, 4, 6 and 8). Thus, Rap1 can block progression of the replication fork in a sequence specific manner and the magnitude of this effect depends on the number of Rap1 molecules bound to the template. Notably, a small fraction of forks bypassed the telomere in the presence of Rap1, but this fraction increased only modestly over 2 h, suggesting the replication block is relatively stable (Supplementary Figure S3).

### Lagging strand telomere replication is inhibited by Rap1

**Figure 4.**
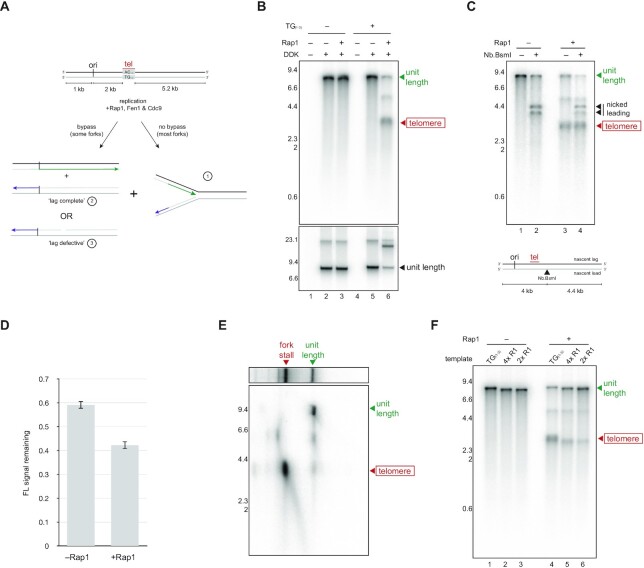
Rap1 is inhibitory for lagging strand replication behind the fork. (**A**) Schematic showing the layout of the template, and potential outcomes of replication in the presence of Rap1. Most forks are expected to stall on the telomere due to the roadblock effect of Rap1, giving rise to leading and lagging strand products measuring 3 kb (scenario ‘1’). A fraction of forks bypass the telomere in the presence of Rap1 (left hand side of diagram), giving rise to a unit length leading strand product, and lagging strand products that are either unit length if complete (scenario ‘2’), or approximately 5 and 3 kb if there is a defect over telomeric DNA (scenario ‘3’). (**B**) Replication reactions performed with telomeric and non-telomeric templates for 1 h in the presence or absence of Rap1 and DDK as indicated were analysed by denaturing alkaline (top panel) and native (bottom panel) agarose electrophoresis. (**C**) Replication products from reactions performed for 1 h with telomeric template in the presence or absence of Rap1 as indicated were nicked with Nb.BsmI, and analysed by denaturing alkaline agarose electrophoresis. (**D**) Quantification showing the fraction of unit length products remaining after Nb.BsmI treatment of replication products from telomeric or non-telomeric templates in the presence and absence of Rap1. Average of three independent experiments. Error bars show the standard deviation. (**E**) Replication products from a 1 h reaction containing telomeric template and Rap1 were analysed by native agarose electrophoresis (top panel). The lane was then excised and analysed by denaturing alkaline agarose electrophoresis. The position of unit length and stalled fork products in the native dimension are indicated. (**F**) Replication reactions performed for 1 h in the presence or absence of 100 nM Rap1 with the templates indicated were analysed by denaturing alkaline agarose electrophoresis. Templates ‘4× R1’ and ‘2× R1’ contain either four or two consensus Rap1 binding sites in place of telomeric DNA (TG_(1–3)_), which contains seven consensus Rap1 binding sites.

Figure 3 shows that telomere-bound Rap1 can act as a roadblock to the replisome. To determine whether replication events behind the fork are also affected, we analysed lagging strand synthesis in our reactions by adding Fen1 and Cdc9. As described in Figure 2, telomeric lagging strand defects in the presence of Fen1 and Cdc9 should give rise to two radiolabelled products measuring approximately 5 and 3 kb in addition to leading strand products that are unit length (Figure 4A, scenario 3). Since most forks stall on the telomere in the presence of Rap1 (Figure 3), we also expect a prominent 3 kb product when Rap1 and telomeric DNA are present, irrespective of whether lagging strand synthesis is complete (Figure 4A, scenario 1). Figure 4B shows that Rap1 had no effect on the replication of a control template in the presence of Fen1 and Cdc9 (compare lanes 2 and 3), but caused the appearance of bands at 3 and 5 kb when a telomeric template was used (compare lanes 5 and 6). In combination with restriction enzyme mapping that confirms the identity of 3 and 5 kb products (Supplementary Figure S4), this result suggests Rap1 is inhibitory for lagging strand replication of telomeric DNA behind the fork. To examine this idea, we analysed the lagging strand directly by treating purified replication products with Nb.BsmI to nick the nascent leading strand. Consistent with the data in Figure 4B, the fraction of unit length products resistant to Nb.BsmI was reduced by approximately a third when Rap1 was added, suggesting lagging strand replication by the rightward fork (which copies the telomere) was indeed defective (Figure 4C and D).

Interestingly, the size of the 5 kb fragment indicates that lagging strand telomeric defects may persist even after replication forks have reached the template end (Figure 4B, scenario 3). To examine this idea, we used 2D native-denaturing electrophoresis to distinguish completely replicated DNA molecules (which run at unit length in the native dimension) from those that contain stalled or paused forks (which migrate more slowly). Figure 4E shows that most products measuring 3 kb were associated with molecules migrating more slowly than unit length in the native dimension, consistent with telomere stalled replication forks. In contrast, molecules running at unit length were composed of strands measuring not only unit length, but also 3 and 5 kb. Thus, telomeric gaps or nicks induced by Rap1 can persist when replication is otherwise complete. We note that some products measuring ∼5 kb migrate more slowly than unit length in these assays. The reason for this is currently unclear but may reflect the formation of strand displaced or structured lagging strand intermediates in the presence of Rap1. As in Figure 3, the inhibitory effect of Rap1 on the lagging strand was reduced when the template contained fewer Rap1 binding sites (Figure 4F, compare lanes 4–6).

### Pif1 promotes leading and lagging strand replication in the presence of Rap1

**Figure 5.**
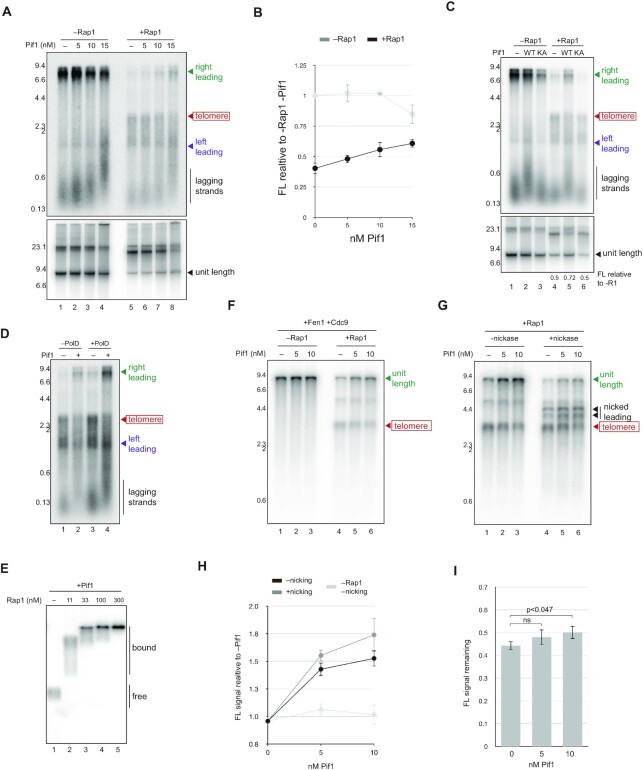
Pif1 promotes leading and lagging strand telomere replication in the presence of Rap1. (**A**) Replication products from reactions performed for 1 h with telomeric template in the presence or absence of 100 nM Rap1 and the Pif1 concentrations indicated were analysed by denaturing (top panel) and native (bottom panel) agarose electrophoresis. (**B**) Quantification showing intensity of unit length bands after native agarose electrophoresis of products from the reactions indicated. Average of three independent experiments. Error bars show standard deviation. (**C**) Replication products from reactions performed for 1 h with telomeric templates in the presence or absence of 100 nM Rap1 with 15 nM wild type or helicase inactive Pif1 as indicated were analysed by denaturing (top panel) and native (bottom panel) agarose electrophoresis. Ratios of unit length products formed in the presence relative to the absence of Rap1 are shown for each Pif1 condition analysed. (**D**) Replication reactions containing telomeric template and 100 nM Rap1 were performed for 1 h in the presence and absence of polymerase δ and 15 nM Pif1 as indicated. Reaction products were analysed by denaturing alkaline electrophoresis. (**E**) EMSA assay performed as in Figure 3A, except 15 nM Pif1 was included. (**F**) in vitro replication reactions with telomeric template and Fen1/Cdc9 were performed for 1 h in the presence or absence of 100 nM Rap1 and the Pif1 concentrations indicated. Reaction products were analysed by denaturing alkaline electrophoresis. (**G**) Replication products from part f were purified, nicked with Nb.BsmI and analysed by denaturing alkaline electrophoresis. Unit length products with and without nicking were quantified, and the average intensity from three independent experiments is presented in (**H**) error bars show standard deviation. (**I**) The ratio of unit length products with or without nicking with Nb.BsmI was calculated from three independent experiments. Error bars show standard deviation. Statistical significance was calculated with Student's *t*-test.

Rap1 is therefore inhibitory for two distinct steps in the replication process: replisome progression (Figure 3), and lagging strand replication behind the fork (Figure 4). We hypothesised that if Rap1 is a major cause of telomeric replication stress *in vivo*, factors known to promote telomere replication within cells may prevent or attenuate these effects. Pausing of replication forks at budding yeast telomeres is increased without the Pif-family helicase Rrm3 (3). We were unable to produce sufficient amounts of helicase active Rrm3, but were able to express and purify the only other Pif-family helicase in budding yeast, Pif1. Titration of Pif1 into replication reactions caused the length and intensity of OFs to increase (Figure 5A, lanes 1–4), consistent with enhanced strand displacement synthesis by pol δ (32). However, in reactions containing Rap1, Pif1 also increased the number of forks able to bypass the telomere and reach the template end (Figure 5A, lanes 5–8 and Figure 5B). Previous work has found that ATP-dependent translocation of Pif1 along single stranded DNA is able to remove even tightly bound proteins (33). To examine whether the helicase activity of Pif1 is required for this bypass effect, we purified helicase inactive Pif1 in which lysine 264 has been substituted for alanine (34). Replication was slightly reduced in the presence of this mutant (Figure 5C, lane 3); nonetheless, when Rap1 was added, helicase inactive Pif1 did not increase the number of forks that could reach the template end, indicating that DNA unwinding by Pif1 is required for Rap1 bypass to take place (Figure 5C, compare lanes 4–6, and FL fraction noted beneath the bottom panel). Notably, Pif1 could still promote bypass in the absence of Pol δ (Figure 5D), indicating that Pol δ-mediated strand displacement synthesis was not required. Furthermore, as Pif1 had no effect on the equilibrium binding of Rap1 to telomeric DNA (Figure 5E, compare with Figure 3A), we favour a working model for the bypass process in which translocation of Pif1 along single stranded DNA removes Rap1 specifically in front of the replication fork (Supplementary Figure S5a; see discussion).

In reactions containing Fen1 and Cdc9, Pif1 also increased the amount of unit length products formed in the presence of Rap1 (Figure 5F), even when replication products had been treated with Nb.BsmI prior to electrophoresis (Figure 5G and H). Since unit length products resistant to Nb.BsmI should derive mostly from the nascent lagging strand of the rightward fork, these data suggest Pif1 may promote telomeric lagging strand synthesis as well as replisome progression when Rap1 is present. To examine this idea, we calculated the fraction of full length products that were resistant to Nb.BsmI in the presence or absence of Pif1. Figure 5I shows that in reactions containing Rap1, the Nb.BsmI resistant fraction of full length products increased when Pif1 was added, although the difference was only statistically significant with 10 nM Pif1 (Figure 5I). Thus, in addition to enabling the replisome to bypass Rap1, Pif1 may help overcome the inhibitory effect of Rap1 on the telomeric lagging strand.

## DISCUSSION

Semiconservative replication of telomeres ensures that chromosome ends are protected from one cellular generation to the next. Replication forks pause or stall during this process, perhaps helping to preserve telomere integrity during the replication process. Here, we have used an in vitro system for DNA replication to examine the cause of this effect and found that whilst budding yeast telomeric DNA is efficiently replicated in vitro, the telomere binding protein Rap1 inhibits multiple stages of DNA replication and can be partially overcome by Pif1 helicase.

Whilst telomeric DNA was replicated to completion in unperturbed reactions, low concentrations of the G4-stabilizing agent Phen-DC3 were sufficient to induce lagging strand defects over the telomere (Supplementary Figure S2). As Phen-DC3 can act as a general replication inhibitor in vitro (Supplementary Figure S2b), it remains to be determined whether this effect is due to stabilization of G4s on the G-rich lagging strand template or to low levels of replication stress that act synergistically with telomeric DNA. In any case, as the vast majority of replication events on telomeric templates proceed to completion in unperturbed conditions (Figures 1 and 2), any inhibitory effect of G-quadruplexes or secondary structures in general during telomere replication is likely to require factors or components in addition to the replisome. It will be interesting to determine whether this is also the case for other sequences such as trinucleotide or inverted repeats, which have also been proposed to inhibit replication (35), and to examine the effect of telomeric properties such as R-loops, which can promote the assembly of secondary structures on G-rich sequences (36). As replication initiates asynchronously in vitro, a limitation of our study is that we are unlikely to detect transient slowing or pausing of replication that is less pronounced, and alternative approaches such as single molecule imaging may ultimately be required to exhaustively examine the kinetics of replication across specific sequences in vitro.

Our finding that Rap1 is a potent inhibitor of telomere replication is consistent with previous in vivo work showing that tethering of Rap1 to an internal chromosomal site induces replication fork pausing (20) and that budding yeast replication forks pause or stall at internal telomeric DNA tracts that bind Rap1, but not at GC-rich tracts that do not (12). Notably, we show that Rap1 not only acts as a roadblock to the replisome, but also potently inhibits lagging strand replication behind the fork (Figure 4). We suggest two non-exclusive models for how this may take place: first, as protein blocks including Rap1 are known to inhibit Pol δ in simple strand displacement assays (37,38), binding of Rap1 towards the 5′ end of a nascent Okazaki fragment on the telomere could block the final stages of Okazaki fragment processing (Supplementary Figure S5b, model i). Second, as Rap1 can bind single-stranded G-rich telomeric sequences and promote G4 assembly (39,40), Rap1 may inhibit Pol δ by inducing secondary structures on the unwound lagging strand template (Supplementary Figure S5, model ii). Whilst we cannot currently distinguish between these possibilities, artificial Rap1 binding sites that are not G-rich are still able to induce Rap1-dependent lagging strand defects (Figure 4), suggesting G-quadruplexes are not obligatory for the effect we observe.

We have found that the roadblock effect of Rap1 can be partially overcome by Pif1 (Figure 5), a member of the Pif-family helicases that promote telomere replication *in vivo* (3). Since Pif1 does not constitutively remove Rap1 from the template, and is known to translocate on single stranded DNA (33,41), we favour a working model for this process in which Pif1- removes Rap1 in a replication coupled manner (Supplementary Figure S5a). This model is consistent with recent work showing that Pif1 can remove double hexamers of the minichromosome maintenance (MCM) complex from DNA during in vitro replication (26), and the idea that Pif-family helicases work as ‘sweepases’ that generally remove protein blocks in front of the fork (42), a role that is likely to be performed by Rrm3 in vivo (42). The mechanism by which Pif1 promotes lagging strand replication in the face of Rap1 will depend on the nature of the block encountered, but two possible models are presented in Supplementary Figure S5b. Notably, Rap1 is only partially overcome by Pif1 *in vitro*, perhaps indicating that a factor or component is missing or that the conditions in the assay are suboptimal. However, since replication pauses at telomeres even in wild type cells (3), an alternative possibility is that arrays of Rap1 bound to each budding yeast telomere (43) are a particularly challenging barrier for Pif1 or Rrm3 to remove. Indeed, tracts of Rap1 binding sites are converted into double strand breaks in a replication-dependent manner *in viv*o, indicating that at least a proportion of forks ‘stuck’ behind Rap1 are processed and cleaved (20).

There are significant differences in the sequences and proteins at telomeres in yeast and human cells. However, like yeast Rap1, TRF1 and TRF2 use Myb-type domains to bind human telomeric DNA (44), and RTEL1, which promotes telomere replication in human cells (8) can remove protein blocks from in front of the replication fork (45). It is interesting to consider that in addition to facilitating replication by recruiting accessory factors to DNA (5), the hundreds of shelterin molecules bound to each human telomere (46) may also prove inhibitory in a manner analogous to budding yeast Rap1. High concentrations of TRF1 and TRF2 can block replication orchestrated by the viral helicase large T-antigen, in line with this idea (11). However, as large T-antigen is heterologous to the CMG helicase and the impact of shelterin on lagging strand replication is yet to be examined, further work is required to determine whether this is indeed the case.

## DATA AVAILABILITY

All data are available in the main figures and supplementary data file.

## Supplementary Material

gkab416_Supplemental_File

## References

[1] Maciejowski J. , de Lange T. Telomeres in cancer: tumour suppression and genome instability. Nat. Rev. Mol. Cell Biol. 2017; 18:175–186. 28096526 10.1038/nrm.2016.171 PMC5589191

[2] Sabatier L. , Ricoul M., Pottier G., Murnane J.P. The loss of a single telomere can result in instability of multiple chromosomes in a human tumor cell line. Mol. Cancer Res. 2005; 3:139–150. 15798094 10.1158/1541-7786.MCR-04-0194

[3] Ivessa A.S. , Zhou J.Q., Schulz V.P., Monson E.K., Zakian V.A. Saccharomyces Rrm3p, a 5′ to 3′ DNA helicase that promotes replication fork progression through telomeric and subtelomeric DNA. Genes Dev. 2002; 16:1383–1396. 12050116 10.1101/gad.982902 PMC186315

[4] Miller K.M. , Rog O., Cooper J.P. Semi-conservative DNA replication through telomeres requires Taz1. Nature. 2006; 440:824–828. 16598261 10.1038/nature04638

[5] Sfeir A. , Kosiyatrakul S.T., Hockemeyer D., MacRae S.L., Karlseder J., Schildkraut C.L., de Lange T. Mammalian telomeres resemble fragile sites and require TRF1 for efficient replication. Cell. 2009; 138:90–103. 19596237 10.1016/j.cell.2009.06.021 PMC2723738

[6] Crabbe L. , Verdun R.E., Haggblom C.I., Karlseder J. Defective telomere lagging strand synthesis in cells lacking WRN helicase activity. Science. 2004; 306:1951–1953. 15591207 10.1126/science.1103619

[7] McDonald K.R. , Sabouri N., Webb C.J., Zakian V.A. The Pif1 family helicase Pfh1 facilitates telomere replication and has an RPA-dependent role during telomere lengthening. DNA Repair (Amst.). 2014; 24:80–86. 25303777 10.1016/j.dnarep.2014.09.008 PMC4252562

[8] Vannier J.B. , Pavicic-Kaltenbrunner V., Petalcorin M.I., Ding H., Boulton S.J. RTEL1 dismantles T loops and counteracts telomeric G4-DNA to maintain telomere integrity. Cell. 2012; 149:795–806. 22579284 10.1016/j.cell.2012.03.030

[9] Mendez-Bermudez A. , Giraud-Panis M.J., Ye J., Gilson E. Heterochromatin replication goes hand in hand with telomere protection. Nat. Struct. Mol. Biol. 2020; 27:313–318. 32231287 10.1038/s41594-020-0400-1

[10] Luke B. , Lingner J. TERRA: telomeric repeat-containing RNA. EMBO J. 2009; 28:2503–2510. 19629047 10.1038/emboj.2009.166 PMC2722245

[11] Ohki R. , Ishikawa F. Telomere-bound TRF1 and TRF2 stall the replication fork at telomeric repeats. Nucleic Acids Res. 2004; 32:1627–1637. 15007108 10.1093/nar/gkh309 PMC390322

[12] Makovets S. , Herskowitz I., Blackburn E.H. Anatomy and dynamics of DNA replication fork movement in yeast telomeric regions. Mol. Cell. Biol. 2004; 24:4019–4031. 15082794 10.1128/MCB.24.9.4019-4031.2004 PMC387773

[13] Gilson E. , Geli V. How telomeres are replicated. Nat. Rev. Mol. Cell Biol. 2007; 8:825–838. 17885666 10.1038/nrm2259

[14] Tran P.L. , Mergny J.L., Alberti P. Stability of telomeric G-quadruplexes. Nucleic Acids Res. 2011; 39:3282–3294. 21177648 10.1093/nar/gkq1292 PMC3082875

[15] Woodford K.J. , Howell R.M., Usdin K. A novel K(+)-dependent DNA synthesis arrest site in a commonly occurring sequence motif in eukaryotes. J. Biol. Chem. 1994; 269:27029–27035. 7929444

[16] Jurikova K. , Gajarsky M., Hajikazemi M., Nosek J., Prochazkova K., Paeschke K., Trantirek L., Tomaska L. Role of folding kinetics of secondary structures in telomeric G-overhangs in the regulation of telomere maintenance in *Saccharomyces cerevisiae*. J. Biol. Chem. 2020; 295:8958–8971. 32385108 10.1074/jbc.RA120.012914 PMC7335780

[17] Biffi G. , Tannahill D., McCafferty J., Balasubramanian S. Quantitative visualization of DNA G-quadruplex structures in human cells. Nat. Chem. 2013; 5:182–186. 23422559 10.1038/nchem.1548 PMC3622242

[18] Lormand J.D. , Buncher N., Murphy C.T., Kaur P., Lee M.Y., Burgers P., Wang H., Kunkel T.A., Opresko P.L. DNA polymerase delta stalls on telomeric lagging strand templates independently from G-quadruplex formation. Nucleic Acids Res. 2013; 41:10323–10333. 24038470 10.1093/nar/gkt813 PMC3905856

[19] Labib K. , Hodgson B. Replication fork barriers: pausing for a break or stalling for time. EMBO Rep. 2007; 8:346–353. 17401409 10.1038/sj.embor.7400940 PMC1852754

[20] Goto G.H. , Zencir S., Hirano Y., Ogi H., Ivessa A., Sugimoto K. Binding of multiple Rap1 proteins stimulates chromosome breakage induction during DNA replication. PLos Genet. 2015; 11:e1005283. 26263073 10.1371/journal.pgen.1005283 PMC4532487

[21] Coster G. , Diffley J.F.X. Bidirectional eukaryotic DNA replication is established by quasi-symmetrical helicase loading. Science. 2017; 357:314–318. 28729513 10.1126/science.aan0063 PMC5608077

[22] Wang S.S. , Zakian V.A. Sequencing of *Saccharomyces* telomeres cloned using T4 DNA polymerase reveals two domains. Mol. Cell. Biol. 1990; 10:4415–4419. 2196453 10.1128/mcb.10.8.4415-4419.1990 PMC361005

[23] Frigola J. , Remus D., Mehanna A., Diffley J.F.X. ATPase-dependent quality control of DNA replication origin licensing. Nature. 2013; 495:339–343. 23474987 10.1038/nature11920 PMC4825857

[24] Yeeles J.T. , Deegan T.D., Janska A., Early A., Diffley J.F.X. Regulated eukaryotic DNA replication origin firing with purified proteins. Nature. 2015; 519:431–435. 25739503 10.1038/nature14285 PMC4874468

[25] Yeeles J.T. , Janska A., Early A., Diffley J.F.X. How the eukaryotic replisome achieves rapid and efficient DNA replication. Mol. Cell. 2017; 65:105–116. 27989442 10.1016/j.molcel.2016.11.017 PMC5222725

[26] Hill J. , Eickhoff P., Drury L.S., Costa A., Diffley J.F.X. The eukaryotic replisome requires an additional helicase to disarm dormant replication origins. 2020; biorxiv doi:17 September 2020, preprint: not peer reviewed 10.1101/2020.09.17.301366.

[27] Deegan T.D. , Baxter J., Ortiz Bazan M.A., Yeeles J.T.P., Labib K.P.M. Pif1-Family helicases support fork convergence during DNA replication termination in eukaryotes. Mol. Cell. 2019; 74:231–244. 30850330 10.1016/j.molcel.2019.01.040 PMC6477153

[28] Douglas M.E. , Ali F.A., Costa A., Diffley J.F.X. The mechanism of eukaryotic CMG helicase activation. Nature. 2018; 555:265–268. 29489749 10.1038/nature25787 PMC6847044

[29] Taylor M.R.G. , Yeeles J.T.P. The initial response of a eukaryotic replisome to DNA damage. Mol. Cell. 2018; 70:1067–1080. 29944888 10.1016/j.molcel.2018.04.022 PMC6024075

[30] Konig P. , Giraldo R., Chapman L., Rhodes D. The crystal structure of the DNA-binding domain of yeast RAP1 in complex with telomeric DNA. Cell. 1996; 85:125–136. 8620531 10.1016/s0092-8674(00)81088-0

[31] Lieb J.D. , Liu X., Botstein D., Brown P.O. Promoter-specific binding of Rap1 revealed by genome-wide maps of protein-DNA association. Nat. Genet. 2001; 28:327–334. 11455386 10.1038/ng569

[32] Pike J.E. , Burgers P.M., Campbell J.L., Bambara R.A. Pif1 helicase lengthens some Okazaki fragment flaps necessitating Dna2 nuclease/helicase action in the two-nuclease processing pathway. J. Biol. Chem. 2009; 284:25170–25180. 19605347 10.1074/jbc.M109.023325 PMC2757220

[33] Ramanagoudr-Bhojappa R. , Chib S., Byrd A.K., Aarattuthodiyil S., Pandey M., Patel S.S., Raney K.D. Yeast Pif1 helicase exhibits a one-base-pair stepping mechanism for unwinding duplex DNA. J. Biol. Chem. 2013; 288:16185–16195. 23596008 10.1074/jbc.M113.470013 PMC3668774

[34] Zhou J. , Monson E.K., Teng S.C., Schulz V.P., Zakian V.A. Pif1p helicase, a catalytic inhibitor of telomerase in yeast. Science. 2000; 289:771–774. 10926538 10.1126/science.289.5480.771

[35] Khristich A.N. , Mirkin S.M. On the wrong DNA track: molecular mechanisms of repeat-mediated genome instability. J. Biol. Chem. 2020; 295:4134–4170. 32060097 10.1074/jbc.REV119.007678 PMC7105313

[36] Carrasco-Salas Y. , Malapert A., Sulthana S., Molcrette B., Chazot-Franguiadakis L., Bernard P., Chedin F., Faivre-Moskalenko C., Vanoosthuyse V. The extruded non-template strand determines the architecture of R-loops. Nucleic Acids Res. 2019; 47:6783–6795. 31066439 10.1093/nar/gkz341 PMC6648340

[37] Koc K.N. , Singh S.P., Stodola J.L., Burgers P.M., Galletto R. Pif1 removes a Rap1-dependent barrier to the strand displacement activity of DNA polymerase delta. Nucleic Acids Res. 2016; 44:3811–3819. 27001517 10.1093/nar/gkw181 PMC4856994

[38] Sparks M.A. , Burgers P.M., Galletto R. Pif1, RPA, and FEN1 modulate the ability of DNA polymerase delta to overcome protein barriers during DNA synthesis. J. Biol. Chem. 2020; 295:15883–15891. 32913126 10.1074/jbc.RA120.015699 PMC7681027

[39] Giraldo R. , Rhodes D. The yeast telomere-binding protein RAP1 binds to and promotes the formation of DNA quadruplexes in telomeric DNA. EMBO J. 1994; 13:2411–2420. 8194531 10.1002/j.1460-2075.1994.tb06526.x PMC395107

[40] Traczyk A. , Liew C.W., Gill D.J., Rhodes D. Structural basis of G-quadruplex DNA recognition by the yeast telomeric protein Rap1. Nucleic Acids Res. 2020; 48:4562–4571. 32187364 10.1093/nar/gkaa171 PMC7192608

[41] Galletto R. , Tomko E.J. Translocation of *Saccharomyces cerevisiae* Pif1 helicase monomers on single-stranded DNA. Nucleic Acids Res. 2013; 41:4613–4627. 23446274 10.1093/nar/gkt117 PMC3632115

[42] Ivessa A.S. , Lenzmeier B.A., Bessler J.B., Goudsouzian L.K., Schnakenberg S.L., Zakian V.A. The *Saccharomyces cerevisiae* helicase Rrm3p facilitates replication past nonhistone protein–DNA complexes. Mol. Cell. 2003; 12:1525–1536. 14690605 10.1016/s1097-2765(03)00456-8

[43] Gilson E. , Roberge M., Giraldo R., Rhodes D., Gasser S.M. Distortion of the DNA double helix by RAP1 at silencers and multiple telomeric binding sites. J. Mol. Biol. 1993; 231:293–310. 8510148 10.1006/jmbi.1993.1283

[44] Konig P. , Fairall L., Rhodes D. Sequence-specific DNA recognition by the myb-like domain of the human telomere binding protein TRF1: a model for the protein-DNA complex. Nucleic Acids Res. 1998; 26:1731–1740. 9512546 10.1093/nar/26.7.1731 PMC147458

[45] Sparks J.L. , Chistol G., Gao A.O., Raschle M., Larsen N.B., Mann M., Duxin J.P., Walter J.C. The CMG helicase bypasses DNA-protein cross-links to facilitate their repair. Cell. 2019; 176:167–181. 30595447 10.1016/j.cell.2018.10.053 PMC6475077

[46] Erdel F. , Kratz K., Willcox S., Griffith J.D., Greene E.C., de Lange T. Telomere recognition and assembly mechanism of mammalian shelterin. Cell Rep. 2017; 18:41–53. 28052260 10.1016/j.celrep.2016.12.005 PMC5225662

