# Supplementary material for: Budding yeast Rap1, but not telomeric DNA, is inhibitory for multiple stages of DNA replication in vitro
Source: Nucleic Acids Res. 2021 May 28;49(10):5671–83. doi: 10.1093/nar/gkab416 (PMC8191780; doi:10.1093/nar/gkab416)
Supplement: gkab416_Supplemental_File [file gkab416_supplemental_file.pdf]

Budding yeast Rap1, but not telomeric DNA, is inhibitory for multiple stages of DNA replication in vitro.

Max E. Douglas and John F.X. Diffley

Supplementary information.

Sequence of DNA constructs used.

MD154:

TCGCGCGTTTCGGTGATGACGGTGAAAACCTCTGACACATGCAGCTCCCGGAGACGGT
CACAGCTTGTCTGTAAGCGGATGCCGGGAGCAGACAAGCCCGTCAGGGCGCGTCAGC
GGGTGTTGGCGGGTGTCGGGGCTGGCTTAACTATGCGGCATCAGAGCAGATTGTACT
GAGAGTGCACCATATGCGGTGTGAAATACCGCACAGATGCGTAAGGAGAAAATACCGC
ATCAGGCGCCATTCGCCATTCAGGCTGCGCAACTGTTGGGAAGGGCGATCGGTGCGG
GCCTCTTCGCTATTACGCCAGCTGGCGAAAGGGGGATGTGCTGCAAGGCGATTAAGTT
GGGTAACGCCAGGGTTTTCCCAGTCACGACGTTGTAAAACGACGGCCAGTGAATTCCT
CGATTTTTTTATGTTTAGTTTCGCGGACGACGGTTTCGAGGTGGCGGTCTGGACCACGC
CGGAGAGCGTCGAAGCGGAGGCGGTGTTCGCCGAGATCGGCTCGCGCAAAGCCGAG
TTGAGCGAACTAAACATAAAAATACAGCATCAGATGGTAGGCCTCCTGGCGCCGCACC
GGCCTCAGCATCCGGTACCTCAGCTGGCCACCGTCGGCGTCTCGCACGACCACCAGT
GCAAGGGTCTGAGCAGCGCCGTCGTGCTCCTCGGAGTGGAGGCAGCCGAGCGCGAC
GGTGTGCCCGCCTTCCTGGAGACCTCCGCGCTCCGCAACCTCCACTTCTACGAGCGG
CTCGGCTTCACCGTCACCGCCGACGTCGAGGTGCCCGAAGGACCGCGCACCTGGTGC
ATGACCCGCAAGCCCGGTGCCTGACGCTCGCCACACGACCCGCAGCGCCCGACCGAA
AGGAGCGCACGACCCGGGTTAACCTAGACAATGGAAGTACCTATGTTATTGTCGAACC
AGTTTCTGGAAAAGGTACAGTCAACATCATTTCCGGTAACCTATACTTACACTACCCTGA
CACATTTACTGGCCAAACTGTTGTATTCAAGGGTGAAGGTGTCCTTGCTGTTGACCCAA
CCGAAACCAATGCCACTCCTATTCCTGTTGTTGGCTACACTGGTAAGAACCAAATTGCC
ATTACCGCTGACATCACTGCTCTTTCTTATGACGGTACTACCGGTGTTCTAACTGCAAC
CCAAGGTAACAGACAATTCTCTTTTGCTATTGGTACCGGATTCTCCAGCTCTGACTTCA
GCGTCTCTGAAGGAATCTTTGCAGGTGCTTACGCTTACTACCTAAACTACAATGGTGTT
GTCGCTACTAGTGCCGCTTCTTCATCCACTGCATCTGGTGCTTCCGCTTCCGTTACCGG
TTCTACTTCATTCGGTGCTTCCGTTACCGGTTCAACTGCTTCCACTTCATTCGGTGCTTC
CGTTACCGGTTCAACTGCTTCCACTTCATTTGGTGCTTCCGTTACCGGTTCAACATCTGT
TTACACTACAACACTAGACTATGTAAATGCCACAAGCACAGTCGTAGTTTCTTGTTCAGA
GACAACTGACTCCAATGGTAACGTCTATACCATTACCACAACCGTTCCATGCTCATCCA
CTACCGCCACTATTACTTCTTGTGATGAAACTGGATGTCACGTTAGTACATCAACCGGT
GCTGTTGTAACTGAAACCGTTTCTTCCAAATCATACACAACTGCCACTGTAACTCACTGT
GACGATAATGGCTGTAACACCAAGACTGTCACTTCTGAATGTTCCAAAGAAACATCAGC
AACAACTGCTTCTCCAAAATCATACACCACTGTCACCGTAACCCACTGTGACGACAATG
GCTGTAACACCAAGACTGTCACCTCTGAAGCTCCTGAAGCTACCACCACAACTACTGTT
TCTTCTCAATCGTACACCACTGCCACCGTCACCCACTGTGATGACAATGGATGTAAGAC
CAAGACTGTCACTTCTGAAGCTCCTGAAGCCACAACCACTACTGTTTCTCCAAAGACAT
ACACCACCGCTACTGTTACTCAGTGTGATGACAATGGATGTAGCACCAAGACTGTCACT
TCTGAATGTCCTGAAGAAACTTCAGCAACTACTACTTCTCCAAAATCATACACTACTGTT
ACCGTTACTCACTGTGACGACAACGGCTGTAACACTAAGACTGTCACCTCTGAGGCCC
CTGAAGCCACAACCACTACTGTTTCTCCAAAGACATACACCACCGCTACTGTTACTCAG
TGCGATGACAATGGATGTAGCACCAAGACTGTCACTTCTGAAGCTCCTAAAGAAACTTC
AGAAACTTCAGAAACCAGTGCTGCCCCTAAGGACATACACTACTGCCACTGGTTACTCA
ATGGTGATGACAATGGTTGTAACGTCAAGATAATCACCTCTAAAATACCTGAAGCTACTT
CAACCGTCACGCAACTAGTGCTTCTCCAAAGTCATACACTACTGTCACTTCTGAGGGTT
CTAAAGCAACCTCATTGAGTCGACGCGGCCGCCATGGTTAATTAAGCTAGCACGACTA
CGCATCCCTCTGGGCCTTACATAGCCGGATACAGTGACTTTGACAGGTTTGCGGGGCA
CAGCAATGACTTGCATAGCTGCGTGCGGGGGAAGGAACTCTTGCGTGTGAGTATGTAG

ACCCCTGTACTACGGATGCGGGCAGAAGATGTGGGCAGAGACACCCGCGTCAAGTTC
TCGACCTTCCCGTGGGAGGTGTTCCAGTCCGCCATACGACCATACCGTTCGGGCATGG
CACTATGTACGCCGTCCCCATTCTGGTAGCCATCATCCCTATCACGGCTTCGAGCGAGT
GGTGACGGATATTCCCCACGGACGGACATCCTACTCACAGTCGGTCACATTGGGGTAC
TCCTTGGCTCTTCCGCTTGGCCCGGTCTGTTGGGCCCCCGTAGCGTGAGTTTCGGCCC
CGCGCTGCCCAGTGTCGGCCATTCTCATCGGGGCCCCACTTCTGGATACCCCGACCTA
TTTTGACGGCACCATTGGCGGAAGTTGCTGGACCTGCTCACCGTGGAGTCCTCCGCAG
GCCTCCCTCCTACATTAGACGATAAGCTCCGGCAAGCAGCATTGATCAACGCAAGGAT
CGGCGATGTAGACAGAGAGACGGCTGATTACCCTTGTTGGTGTGGTATCGCTAAGCTG
CGCCGCGGAGCCTTATGGCATAGCCGTCCGCGGAGCATTCCGGTAGCGCTTATGGTC
CATAGCACGTCCATCGCATCCGGGCGTGCGCTCTGCTTGACGACCCCTTGGCGCAGA
GGTGCTGGCCGCGTGCTAAGTTGAAGCGGCTGCACTGCTGCAAGGTCCGTCACGGAG
GCGTCGGACCGGCAGGAGCACTAGCCCATCGACCCGTACGGGAACACTCTATATCGC
TCTCGGACGGACAGATTACTAGAGTGCCGCTTTCAGCCCCCCTGTCGTCGCCGAGGTG
TGTAGCATGGCGATGTCGTGGTTCCACTCTATCGGGGCATCAGCTGATGCGGAGGGAG
ATCCGGAATGAACCGGCCTATGTCACAGAAACTGTGCAAACACCCGATGTCGTTAGTG
TAGGTTCCGACCGATACGTGCTTCGTTGAGAACCCACAATCTGACAACTGGGGACATA
AACCCTACGCCCATCATCTACTGGCGTCCCTGAGGCTCCAGTTCATGTGGTGGGAGAG
CACCCGCCGCAAGGTCTAGTGCAATGGTGGTATGGTAAGCTCGTACCGTGGTAGAGG
CGGCACGGGTAGGACCATCAGTAGTAGGGATAGTGGGAAAGCTCACAGACCACCGCC
TATAGGGGGTGCTTACCTCTACAACAAGCGACTGCCAGTGTAACCCCACGAGCATCGG
AGAAGTCGAACCGACCCGGTCCAGCCGGAGGGACGGGCCTCAAAGCCTCGTCACGAC
GGCTGTCGGCCCGTAACAGAAACCCCGGAGTGAGCTGCCGTGGGCCTGGATAGAACA
GCCCTGGTGGGCCCCATCAGCGGCCCGAACACGTCGCTCTTCGGGACGCGGGCCGA
GGGGCGATGCCTTCCGCTAATCGAGGCCGTTCGTCGATACTTGTTGCGTTCCTAGCGC
CTATAGATGTCTCTCTGCCGGCTCATGTGGACAAGCACAGCATAGCCATTTGTCGGGG
CGCCTCGGTACACGGTATGGGCAGGCGCCTCGTGAGGCCATCGCGTATACCAGGTGT
CCTGTAAGCAGCGAAGGCCCGCACGCGAGATACACTGCCGGGGAACCGCGTGTCCAC
GAGCCGTGGTGGATTTAATCTGGCCGAGGTGTGGACATTCCAGGCGGTGCGTCTGCC
GCCGGGCCCCTCTGGTGACGCACCAGCAGCTCTCCTCCCCAGCGGTCTGGTCTATGG
AAACTACAGGACTAACCTTCCTGGCAACCGGGGGCTGGGAATCCGTGACGTGAGTCAA
GGTATTTGCCCGGTAATCTATGCCCCAGGCATCTAACTATTCCCACTGCCTTGAGGGG
GCCTGCGCTTTCTGCCCGTCGATCCGTAGGACTCGTGCCAACGCGCAGGCTTAGTTCG
AGGTGAAATGTCCGGGGCCAGAGACAACCAGCATCTCGCGTCCTGCCCAACCCCCCT
ACACGCTGTTATAGGCAATCAGCGGGAACCCGGCGCCAGGCGATGGAACGACCTGAA
GTCGGGCAGGGACTTACAGGGAACGTATGTACAACGCAATGAAGCTGGAGGATTGGC
GTGGGAATCGTGCCTCTGTCTAAGCAAGAATGGGCACGGGGTGGCAACCGTCCCCCT
AGCGTGCGGGGTGCACATCGTAACGATCTGGGGGTCGGCAGACTCGCTGTGTTCGGA
ATTTCCCTTTAAGCGCGGGTATTGAGCCAGGCTTATGCCCAGGATCGTAGCAAGCTGA
CCCATACAAGATGTATGCTGCCCGCGTTGCAGACGAGACCAGTCGGAGGTTACGGAG
CATACTAACACGTGGGCGGCCACTGGTGAGCTGCTGCACCCCTGCGGCAACGTTGAA
GCCCCTAAGTTGCTCTGGCTGGACCCAAGCCGTAACACCCGTCTCACTTCATAACCGTT
TGCAATTCACGGCTTGACCTAGACCGGATTGCCATTCTCTCGAAGTAATGTGCAGGCC
GGCGTGCGCGTCCCATGCATACCTGTCATTACTTACCTGAGACTACTCGGAAGTGTGG
CTAGATCTATGCCCACGCGCCTAGTCGGCCCACGCTTGGTTTTTAGGACCCGATGATC
TCCGAGACGCTGCAAGATTCCCAACCTGCCTCACAAGGCGCTGGGTCCTACTCCAGCG
GGACCTAGTGTCTCAAGACGATGAGAGGAGCACTCGTCAGTGGCGTAATCATGGTCAT
AGCTGTTTCCTGTGTGAAATTGGCATGCAAGCTTCTCATCCGCTTCTACTGCCTCCGGT
TCTGCCACTTCCAATTCCTTGAGTTCCATTACTTCTTCAGCATCTAGTGCAAGCGCAACT
GCTTCCAACTCCCTTTCTTCCAGCGATGGTACTATCTATCTACCAACTACTACAATCAGT
GGTGATCTAACTCTTACTGGTAAAGTAATTGCAACAGAAGGTGTTGTGGTCGCAGCTGG
TGCCAAATTGACTCTACTTGACGGTGACAAATATTCTTTCTCAGCTGACCTAAAAGTCTA
CGGTGACTTGCTTGTGAAAAAGTCCAAAGAAACCTATCCAGGTACCGAATTCGACATCT
CCGGTGAAAACTTTGACGTGACCGGTAACTTCAACGCTGAAGAATCCGCTGCCACCTC
TGCATCCATCTACTCCTTCACTCCAAGTTCTTTTGACAACAGTGGTGACATTTCCTTAAG

TCTATCAAAGTCCAAGAAGGGTGAAGTCACTTTCTCTCCATACTCCAATTCTGGTGCCTT
CTCTTTCTCGAACGCTATTCTCAACGGTGGTTCTGTTTCCGGTCTACAACGTAAGCTTG
GCGTAATCATGGTCATAGCTGTTTCCTGTGTGAAATTGTTATCCGCTCACAATTCCACAC
AACATACGAGCCGGAAGCATAAAGTGTAAAGCCTGGGGTGCCTAATGAGTGAGCTAAC
TCACATTAATTGCGTTGCGCTCACTGCCCGCTTTCCAGTCGGGAAACCTGTCGTGCCA
GCTGCATTAATGAATCGGCCAACGCGCGGGGAGAGGCGGTTTGCGTATTGGGCGCTC
TTCCGCTTCCTCGCTCACTGACTCGCTGCGCTCGGTCGTTCGGCTGCGGCGAGCGGTA
TCAGCTCACTCAAAGGCGGTAATACGGTTATCCACAGAATCAGGGGATAACGCAGGAC
AGAACATGTGAGCAGAAGGCCAGCACGAGGCCAGGAGCCGTAAGAAGGCCGCGTTGC
TGGCGTTTTTCCATAGGCTCCGCCCCCCTGACGAGCATCACAAAAATCGACGCTCAAG
TCAGAGGTGGCGAAACCCGACAGGACTATAAAGATACCAGGCGTTTCCCCCTGGAAGC
TCCCTCGTGCGCTCTCCTGTTCCGACCCTGCCGCTTACCGGATACCTGTCCGCCTTTCT
CCCTTCGGGAAGCGTGGCGCTTTCTCATAGCTCACGCTGTAGGTATCTCAGTTCGGTG
TAGGTCGTTCGCTCCAAGCTGGGCTGTGTGCACGAACCCCCCGTTCAGCCCGACCGCT
GCGCCTTATCCGGTAACTATCGTCTTGAGTCCAACCCGGTAAGACACGACTTATCGCCA
CTGGCAGCAGCCACTGGTAACAGGATTAGCAGAGCGAGGTATGTAGGCGGTGCTACA
GAGTTCTTGAAGTGGTGGCCTAACTACGGCTACACTAGAAGAACAGTATTTGGTATCTG
CGCTCTGCTGAAGCCAGTTACCTTCGGAAAAAGAGTTGGTAGCTCTTGATCCGGCAAA
CAAACCACCGCTGGTAGCGGTGGTTTTTTTGTTTGCAAGCAGCAGATTACGCGCAGAA
AAAAAGGATCTCAAGAAGATCCTTTGATCTTTTCTACGGGGTCTGACGCTCAGTGGAAC
GAAAACTCACGTTAAGGGATTTTGGTCATGAGATTATCAACAAGGATCTTCACCTAGAT
CCTTTTAGATTACAGATGAAGTTTTACATCAATCTAGAGTATCTATGAGTAGACTTGGTC
TGACAGTTACCAATGCTTAATCAGTGAGGCACCTATCTCAGCGATCTGTCTATTTCGTTC
ATCCATAGTTGCCTGACTCCCCGTCGTGTAGATAACTACGATACGGGAGGGCTTACCAT
CTGGCCCCAGTGCTGCAATGATACCGCGAGACCCACGCTCACCGGCTCCAGATTTATC
AGCAATAAACCAGCCAGCCGGAAGGGCCGAGCGCAGAAGTGGTCCTGCAACTTTATCC
GCCTCCATCCAGTCTATTAATTGTTGCCGGGAAGCTAGAGTAAGTAGTTCGCCAGTTAA
TAGTTTGCGCAACGTTGTTGCCATTGCTACAGGCATCGTGGTGTCACGCTCGTCGTTTG
GTATGGCTTCATTCAGCTCCGGTTCCCAACGATCAAGGCGAGTTACATGATCCCCCATG
TTGTGCAAAAAAGCGGTTAGCTCCTTCGGTCCTCCGATCGTTGTCAGAAGTAAGTTGGC
CGCAGTGTTATCACTCATGGTTATGGCAGCACTGCATAATTCTCTTACTGTCATGCCATC
CGTAAGATGCTTTTCTGTGACTGGTGAGTACTCAACCAAGTCATTCTGAGAATAGTGTAT
GCGGCGACCGAGTTGCTCTTGCCCGGCGTCAATACGGGATAGTACCGCGCCACATAG
CAGCACTTTGAAAGTGCTCATCATTGGAAAACGTTCTTCGGGGCGAAAACTCTCAAGGA
TCTTACCGCTGTTGAGATCCAGTTCGATGTAACCCACTCGTGCACCCAACTGATCTTCA
GCATCTTTTACTTTCACCAGCGTTTCTGGGTGAGCAAGAACAGGAAGGCACAATGCCG
CAGACAAGGGAATAAGGGCGACACGGAAATGTTGAATACTCATACTCTTCCTGCTTCAA
TAGTATTGAAGCATCTATCAGGGTTAGTGTCTCATGAGCGGATACATATCTGAATGTAT
GTAGAACACTAGACACATAGGGGTTCCGCGCACATTTCCCCGAGAAGTGCCACCTGAC
GTCTAAGACACCAGTATCATCATGACATTGACCTCTAGACATAGGCGTATCACGAGGCC
CTTTCGTC

MD155:
TCGCGCGTTTCGGTGATGACGGTGAAAACCTCTGACACATGCAGCTCCCGGAGACGGT
CACAGCTTGTCTGTAAGCGGATGCCGGGAGCAGACAAGCCCGTCAGGGCGCGTCAGC
GGGTGTTGGCGGGTGTCGGGGCTGGCTTAACTATGCGGCATCAGAGCAGATTGTACT
GAGAGTGCACCATATGCGGTGTGAAATACCGCACAGATGCGTAAGGAGAAAATACCGC
ATCAGGCGCCATTCGCCATTCAGGCTGCGCAACTGTTGGGAAGGGCGATCGGTGCGG
GCCTCTTCGCTATTACGCCAGCTGGCGAAAGGGGGATGTGCTGCAAGGCGATTAAGTT
GGGTAACGCCAGGGTTTTCCCAGTCACGACGTTGTAAAACGACGGCCAGTGAATTCCT
CGATTTTTTTATGTTTAGTTTCGCGGACGACGGTTTCGAGGTGGCGGTCTGGACCACGC
CGGAGAGCGTCGAAGCGGAGGCGGTGTTCGCCGAGATCGGCTCGCGCAAAGCCGAG
TTGAGCGAACTAAACATAAAAATACAGCATCAGATGGTAGGCCTCCTGGCGCCGCACC
GGCCTCAGCATCCGGTACCTCAGCTGGCCACCGTCGGCGTCTCGCACGACCACCAGT
GCAAGGGTCTGAGCAGCGCCGTCGTGCTCCTCGGAGTGGAGGCAGCCGAGCGCGAC

GGTGTGCCCGCCTTCCTGGAGACCTCCGCGCTCCGCAACCTCCACTTCTACGAGCGG
CTCGGCTTCACCGTCACCGCCGACGTCGAGGTGCCCGAAGGACCGCGCACCTGGTGC
ATGACCCGCAAGCCCGGTGCCTGACGCTCGCCACACGACCCGCAGCGCCCGACCGAA
AGGAGCGCACGACCCGGGTTAACCTAGACAATGGAAGTACCTATGTTATTGTCGAACC
AGTTTCTGGAAAAGGTACAGTCAACATCATTTCCGGTAACCTATACTTACACTACCCTGA
CACATTTACTGGCCAAACTGTTGTATTCAAGGGTGAAGGTGTCCTTGCTGTTGACCCAA
CCGAAACCAATGCCACTCCTATTCCTGTTGTTGGCTACACTGGTAAGAACCAAATTGCC
ATTACCGCTGACATCACTGCTCTTTCTTATGACGGTACTACCGGTGTTCTAACTGCAAC
CCAAGGTAACAGACAATTCTCTTTTGCTATTGGTACCGGATTCTCCAGCTCTGACTTCA
GCGTCTCTGAAGGAATCTTTGCAGGTGCTTACGCTTACTACCTAAACTACAATGGTGTT
GTCGCTACTAGTGCCGCTTCTTCATCCACTGCATCTGGTGCTTCCGCTTCCGTTACCGG
TTCTACTTCATTCGGTGCTTCCGTTACCGGTTCAACTGCTTCCACTTCATTCGGTGCTTC
CGTTACCGGTTCAACTGCTTCCACTTCATTTGGTGCTTCCGTTACCGGTTCAACATCTGT
TTACACTACAACACTAGACTATGTAAATGCCACAAGCACAGTCGTAGTTTCTTGTTCAGA
GACAACTGACTCCAATGGTAACGTCTATACCATTACCACAACCGTTCCATGCTCATCCA
CTACCGCCACTATTACTTCTTGTGATGAAACTGGATGTCACGTTAGTACATCAACCGGT
GCTGTTGTAACTGAAACCGTTTCTTCCAAATCATACACAACTGCCACTGTAACTCACTGT
GACGATAATGGCTGTAACACCAAGACTGTCACTTCTGAATGTTCCAAAGAAACATCAGC
AACAACTGCTTCTCCAAAATCATACACCACTGTCACCGTAACCCACTGTGACGACAATG
GCTGTAACACCAAGACTGTCACCTCTGAAGCTCCTGAAGCTACCACCACAACTACTGTT
TCTTCTCAATCGTACACCACTGCCACCGTCACCCACTGTGATGACAATGGATGTAAGAC
CAAGACTGTCACTTCTGAAGCTCCTGAAGCCACAACCACTACTGTTTCTCCAAAGACAT
ACACCACCGCTACTGTTACTCAGTGTGATGACAATGGATGTAGCACCAAGACTGTCACT
TCTGAATGTCCTGAAGAAACTTCAGCAACTACTACTTCTCCAAAATCATACACTACTGTT
ACCGTTACTCACTGTGACGACAACGGCTGTAACACTAAGACTGTCACCTCTGAGGCCC
CTGAAGCCACAACCACTACTGTTTCTCCAAAGACATACACCACCGCTACTGTTACTCAG
TGCGATGACAATGGATGTAGCACCAAGACTGTCACTTCTGAAGCTCCTAAAGAAACTTC
AGAAACTTCAGAAACCAGTGCTGCCCCTAAGGACATACACTACTGCCACTGGTTACTCA
ATGGTGATGACAATGGTTGTAACGTCAAGATAATCACCTCTAAAATACCTGAAGCTACTT
CAACCGTCACGCAACTAGTGCTTCTCCAAAGTCATACACTACTGTCACTTCTGAGGGTT
CTAAAGCAACCTCATTGAGTCGACGCGGCCGCATGGATCCCGGGGTTTTGGGGTTTTG
GGGTTTTGGGGTTTTGGGGTGTGGGTGTGTGTGTGGGTGTGGTGTGTGTGGGTGTGG
TGTGTGGGTGTGGTGTGGTGTGGGTGTGGGTGTGGTGTGGGTGTGGTGTGTGTGGGT
GTGGTGTGTGTGGGTGTGGTGTGTGGTGTGTGTGGGTGTGTGGGTGTGGTGTGTGTG
TGTGTGGGTGTGGTGTGGGTGTGGTGTGTGGGTGTGTGGGTGTGGGTGTGTGTGGTG
TGTGGTGTGTGTGGGTGTGGGTGTGGTGTGTGGGTGTGGGTGTGGTGTGTGGGTGTG
GGTGTGGTGTGTGTGTGTGGGTGTGGTGTGTGGGTGTGGGTGTGGTGTGTGGATGTG
GGGATCCATGTTTAATTAAGCTAGCACGACTACGCATCCCTCTGGGCCTTACATAGCCG
GATACAGTGACTTTGACAGGTTTGCGGGGCACAGCAATGACTTGCATAGCTGCGTGCG
GGGGAAGGAACTCTTGCGTGTGAGTATGTAGACCCCTGTACTACGGATGCGGGCAGAA
GATGTGGGCAGAGACACCCGCGTCAAGTTCTCGACCTTCCCGTGGGAGGTGTTCCAGT
CCGCCATACGACCATACCGTTCGGGCATGGCACTATGTACGCCGTCCCCATTCTGGTA
GCCATCATCCCTATCACGGCTTCGAGCGAGTGGTGACGGATATTCCCCACGGACGGAC
ATCCTACTCACAGTCGGTCACATTGGGGTACTCCTTGGCTCTTCCGCTTGGCCCGGTCT
GTTGGGCCCCCGTAGCGTGAGTTTCGGCCCCGCGCTGCCCAGTGTCGGCCATTCTCA
TCGGGGCCCCACTTCTGGATACCCCGACCTATTTTGACGGCACCATTGGCGGAAGTTG
CTGGACCTGCTCACCGTGGAGTCCTCCGCAGGCCTCCCTCCTACATTAGACGATAAGC
TCCGGCAAGCAGCATTGATCAACGCAAGGATCGGCGATGTAGACAGAGAGACGGCTG
ATTACCCTTGTTGGTGTGGTATCGCTAAGCTGCGCCGCGGAGCCTTATGGCATAGCCG
TCCGCGGAGCATTCCGGTAGCGCTTATGGTCCATAGCACGTCCATCGCATCCGGGCGT
GCGCTCTGCTTGACGACCCCTTGGCGCAGAGGTGCTGGCCGCGTGCTAAGTTGAAGC
GGCTGCACTGCTGCAAGGTCCGTCACGGAGGCGTCGGACCGGCAGGAGCACTAGCC
CATCGACCCGTACGGGAACACTCTATATCGCTCTCGGACGGACAGATTACTAGAGTGC
CGCTTTCAGCCCCCCTGTCGTCGCCGAGGTGTGTAGCATGGCGATGTCGTGGTTCCAC
TCTATCGGGGCATCAGCTGATGCGGAGGGAGATCCGGAATGAACCGGCCTATGTCACA

GAAACTGTGCAAACACCCGATGTCGTTAGTGTAGGTTCCGACCGATACGTGCTTCGTTG
AGAACCCACAATCTGACAACTGGGGACATAAACCCTACGCCCATCATCTACTGGCGTC
CCTGAGGCTCCAGTTCATGTGGTGGGAGAGCACCCGCCGCAAGGTCTAGTGCAATGG
TGGTATGGTAAGCTCGTACCGTGGTAGAGGCGGCACGGGTAGGACCATCAGTAGTAG
GGATAGTGGGAAAGCTCACAGACCACCGCCTATAGGGGGTGCTTACCTCTACAACAAG
CGACTGCCAGTGTAACCCCACGAGCATCGGAGAAGTCGAACCGACCCGGTCCAGCCG
GAGGGACGGGCCTCAAAGCCTCGTCACGACGGCTGTCGGCCCGTAACAGAAACCCCG
GAGTGAGCTGCCGTGGGCCTGGATAGAACAGCCCTGGTGGGCCCCATCAGCGGCCC
GAACACGTCGCTCTTCGGGACGCGGGCCGAGGGGCGATGCCTTCCGCTAATCGAGGC
CGTTCGTCGATACTTGTTGCGTTCCTAGCGCCTATAGATGTCTCTCTGCCGGCTCATGT
GGACAAGCACAGCATAGCCATTTGTCGGGGCGCCTCGGTACACGGTATGGGCAGGCG
CCTCGTGAGGCCATCGCGTATACCAGGTGTCCTGTAAGCAGCGAAGGCCCGCACGCG
AGATACACTGCCGGGGAACCGCGTGTCCACGAGCCGTGGTGGATTTAATCTGGCCGA
GGTGTGGACATTCCAGGCGGTGCGTCTGCCGCCGGGCCCCTCTGGTGACGCACCAGC
AGCTCTCCTCCCCAGCGGTCTGGTCTATGGAAACTACAGGACTAACCTTCCTGGCAAC
CGGGGGCTGGGAATCCGTGACGTGAGTCAAGGTATTTGCCCGGTAATCTATGCCCCAG
GCATCTAACTATTCCCACTGCCTTGAGGGGGCCTGCGCTTTCTGCCCGTCGATCCGTA
GGACTCGTGCCAACGCGCAGGCTTAGTTCGAGGTGAAATGTCCGGGGCCAGAGACAA
CCAGCATCTCGCGTCCTGCCCAACCCCCCTACACGCTGTTATAGGCAATCAGCGGGAA
CCCGGCGCCAGGCGATGGAACGACCTGAAGTCGGGCAGGGACTTACAGGGAACGTAT
GTACAACGCAATGAAGCTGGAGGATTGGCGTGGGAATCGTGCCTCTGTCTAAGCAAGA
ATGGGCACGGGGTGGCAACCGTCCCCCTAGCGTGCGGGGTGCACATCGTAACGATCT
GGGGGTCGGCAGACTCGCTGTGTTCGGAATTTCCCTTTAAGCGCGGGTATTGAGCCAG
GCTTATGCCCAGGATCGTAGCAAGCTGACCCATACAAGATGTATGCTGCCCGCGTTGC
AGACGAGACCAGTCGGAGGTTACGGAGCATACTAACACGTGGGCGGCCACTGGTGAG
CTGCTGCACCCCTGCGGCAACGTTGAAGCCCCTAAGTTGCTCTGGCTGGACCCAAGCC
GTAACACCCGTCTCACTTCATAACCGTTTGCAATTCACGGCTTGACCTAGACCGGATTG
CCATTCTCTCGAAGTAATGTGCAGGCCGGCGTGCGCGTCCCATGCATACCTGTCATTA
CTTACCTGAGACTACTCGGAAGTGTGGCTAGATCTATGCCCACGCGCCTAGTCGGCCC
ACGCTTGGTTTTTAGGACCCGATGATCTCCGAGACGCTGCAAGATTCCCAACCTGCCTC
ACAAGGCGCTGGGTCCTACTCCAGCGGGACCTAGTGTCTCAAGACGATGAGAGGAGC
ACTCGTCAGTGGCGTAATCATGGTCATAGCTGTTTCCTGTGTGAAATTGGCATGCAAGC
TTCTCATCCGCTTCTACTGCCTCCGGTTCTGCCACTTCCAATTCCTTGAGTTCCATTACT
TCTTCAGCATCTAGTGCAAGCGCAACTGCTTCCAACTCCCTTTCTTCCAGCGATGGTAC
TATCTATCTACCAACTACTACAATCAGTGGTGATCTAACTCTTACTGGTAAAGTAATTGC
AACAGAAGGTGTTGTGGTCGCAGCTGGTGCCAAATTGACTCTACTTGACGGTGACAAA
TATTCTTTCTCAGCTGACCTAAAAGTCTACGGTGACTTGCTTGTGAAAAAGTCCAAAGAA
ACCTATCCAGGTACCGAATTCGACATCTCCGGTGAAAACTTTGACGTGACCGGTAACTT
CAACGCTGAAGAATCCGCTGCCACCTCTGCATCCATCTACTCCTTCACTCCAAGTTCTT
TTGACAACAGTGGTGACATTTCCTTAAGTCTATCAAAGTCCAAGAAGGGTGAAGTCACT
TTCTCTCCATACTCCAATTCTGGTGCCTTCTCTTTCTCGAACGCTATTCTCAACGGTGGT
TCTGTTTCCGGTCTACAACGTAAGCTTGGCGTAATCATGGTCATAGCTGTTTCCTGTGT
GAAATTGTTATCCGCTCACAATTCCACACAACATACGAGCCGGAAGCATAAAGTGTAAA
GCCTGGGGTGCCTAATGAGTGAGCTAACTCACATTAATTGCGTTGCGCTCACTGCCCG
CTTTCCAGTCGGGAAACCTGTCGTGCCAGCTGCATTAATGAATCGGCCAACGCGCGGG
GAGAGGCGGTTTGCGTATTGGGCGCTCTTCCGCTTCCTCGCTCACTGACTCGCTGCGC
TCGGTCGTTCGGCTGCGGCGAGCGGTATCAGCTCACTCAAAGGCGGTAATACGGTTAT
CCACAGAATCAGGGGATAACGCAGGACAGAACATGTGAGCAGAAGGCCAGCACGAGG
CCAGGAGCCGTAAGAAGGCCGCGTTGCTGGCGTTTTTCCATAGGCTCCGCCCCCCTGA
CGAGCATCACAAAAATCGACGCTCAAGTCAGAGGTGGCGAAACCCGACAGGACTATAA
AGATACCAGGCGTTTCCCCCTGGAAGCTCCCTCGTGCGCTCTCCTGTTCCGACCCTGC
CGCTTACCGGATACCTGTCCGCCTTTCTCCCTTCGGGAAGCGTGGCGCTTTCTCATAG
CTCACGCTGTAGGTATCTCAGTTCGGTGTAGGTCGTTCGCTCCAAGCTGGGCTGTGTG
CACGAACCCCCCGTTCAGCCCGACCGCTGCGCCTTATCCGGTAACTATCGTCTTGAGT
CCAACCCGGTAAGACACGACTTATCGCCACTGGCAGCAGCCACTGGTAACAGGATTAG

CAGAGCGAGGTATGTAGGCGGTGCTACAGAGTTCTTGAAGTGGTGGCCTAACTACGGC
TACACTAGAAGAACAGTATTTGGTATCTGCGCTCTGCTGAAGCCAGTTACCTTCGGAAA
AAGAGTTGGTAGCTCTTGATCCGGCAAACAAACCACCGCTGGTAGCGGTGGTTTTTTTG
TTTGCAAGCAGCAGATTACGCGCAGAAAAAAAGGATCTCAAGAAGATCCTTTGATCTTT
TCTACGGGGTCTGACGCTCAGTGGAACGAAAACTCACGTTAAGGGATTTTGGTCATGA
GATTATCAACAAGGATCTTCACCTAGATCCTTTTAGATTACAGATGAAGTTTTACATCAA
TCTAGAGTATCTATGAGTAGACTTGGTCTGACAGTTACCAATGCTTAATCAGTGAGGCA
CCTATCTCAGCGATCTGTCTATTTCGTTCATCCATAGTTGCCTGACTCCCCGTCGTGTA
GATAACTACGATACGGGAGGGCTTACCATCTGGCCCCAGTGCTGCAATGATACCGCGA
GACCCACGCTCACCGGCTCCAGATTTATCAGCAATAAACCAGCCAGCCGGAAGGGCC
GAGCGCAGAAGTGGTCCTGCAACTTTATCCGCCTCCATCCAGTCTATTAATTGTTGCCG
GGAAGCTAGAGTAAGTAGTTCGCCAGTTAATAGTTTGCGCAACGTTGTTGCCATTGCTA
CAGGCATCGTGGTGTCACGCTCGTCGTTTGGTATGGCTTCATTCAGCTCCGGTTCCCA
ACGATCAAGGCGAGTTACATGATCCCCCATGTTGTGCAAAAAAGCGGTTAGCTCCTTCG
GTCCTCCGATCGTTGTCAGAAGTAAGTTGGCCGCAGTGTTATCACTCATGGTTATGGCA
GCACTGCATAATTCTCTTACTGTCATGCCATCCGTAAGATGCTTTTCTGTGACTGGTGA
GTACTCAACCAAGTCATTCTGAGAATAGTGTATGCGGCGACCGAGTTGCTCTTGCCCG
GCGTCAATACGGGATAGTACCGCGCCACATAGCAGCACTTTGAAAGTGCTCATCATTG
GAAAACGTTCTTCGGGGCGAAAACTCTCAAGGATCTTACCGCTGTTGAGATCCAGTTCG
ATGTAACCCACTCGTGCACCCAACTGATCTTCAGCATCTTTTACTTTCACCAGCGTTTCT
GGGTGAGCAAGAACAGGAAGGCACAATGCCGCAGACAAGGGAATAAGGGCGACACGG
AAATGTTGAATACTCATACTCTTCCTGCTTCAATAGTATTGAAGCATCTATCAGGGTTAG
TGTCTCATGAGCGGATACATATCTGAATGTATGTAGAACACTAGACACATAGGGGTTCC
GCGCACATTTCCCCGAGAAGTGCCACCTGACGTCTAAGACACCAGTATCATCATGACAT
TGACCTCTAGACATAGGCGTATCACGAGGCCCTTTCGTC.

Supplementary figure legends

Figure S1. Sequence of telomeric DNA analysed in this study. Matches to the consensus Rap1 binding site 5'-(G/A)(G/A)TGN(T/G)(C/T)GG(A/G)T(T/G)(C/T)-3' – are highlighted.

Figure S2. Phen-DC3 can induce sequence-specific defects in lagging strand replication. a. Scans of lanes 3 and 6 from figure 2b **b.** in vitro replication reactions containing Fen1 and Cdc9 were performed for 1 h with control template and the Phen-DC3 concentrations shown, and analysed by alkaline agarose electrophoresis. **c.** replication reactions were carried out as in b., but in the presence or absence of 60 nM Phen-DC3 and the templates indicated. template labelled 'telomere' contains the telomeric insert investigated throughout the manuscript; as shown in the diagram, this insert is composed of 321 bp of budding yeast telomeric DNA ('$TG_{(1-3)}$') and a G-quadruplex prone sequence ('G4') present in the fragment cloned from the original construct generated by Wang and Zakian. Template labelled 'G4' contains only this G4 sequence inserted into the control template. As the intensity

and breadth of the 3 and 5 kb bands was enhanced with the full telomeric insert compared with the G4 motif alone, we conclude that the Phen-DC3 effect with telomeric templates is in part due to telomeric DNA, and in part due to the G4 sequence.

Figure S3. The Rap1 block to DNA replication is largely stable over time. a. Replication reactions containing telomeric template and the Rap1 concentrations indicated were performed for 15, 60 or 120 minutes and replication products analysed by native agarose electrophoresis **b.** unit length products from part a. were quantified and plotted. The change in unit length signal over time in the presence of 100 nM Rap1 is shown in part **c.**

Figure S4. Restriction enzyme analysis of replication products in the presence of Rap1, Fen1 and Cdc9. Replication reactions with telomeric or non-telomeric templates were performed for 1 h in the presence of 100 nM Rap1; replication products were purified, digested with HpaI or AflII restriction enzymes as indicated, and analysed by denaturing alkaline electrophoresis.

Figure S5. Models of the impact of Rap1 and Pif1 on the replication of telomeric DNA. a. working model for leading strand replication. Rap1 acts as a roadblock that prevents replication fork progression at the telomere. The helicase activity of Pif1 promotes Rap1 bypass by the replisome. A potential model for the mechanism is that Pif1 engages the non-translocation strand for CMG and translocates 5'-3 to remove Rap1 from in front of the fork. **b.** model for lagging strand replication. Rap1 may bind towards the 5' end of nascent OFs, blocking strand displacement synthesis by Pol delta (model 'i'), or promote the assembly of G4 structures on the lagging strand template (model 'ii'), blocking pol delta mediated synthesis. Pif1 may over these defects by engaging the 5' flap, or the exposed lagging strand template, translocating in a 5'-3' direction.

```
1   TGTGGGTGTGTGTGTGGGTGTGGTGTGTGTGGGTGTGGTGTGTGGGTGTG
51  GTGTGGTGTGGGTGTGGGTGTGGTGTGGGTGTGGTGTGTGTGGGTGTGGT
101 GTGTGTGGGTGTGGTGTGTGGTGTGTGTGGGTGTGTGGGTGTGGTGTGTG
151 TGTGTGTGGGTGTGGTGTGGGTGTGGTGTGTGGGTGTGTGGGTGTGGGTG
201 TGTGTGGTGTGTGGTGTGTGTGGGTGTGGGTGTGGTGTGTGGGTGTGGGT
251 GTGGTGTGTGGGTGTGGGTGTGGTGTGTGTGTGTGGGTGTGGTGTGTGGG
301 TGTGGGTGTGGTGTGTGGATGTGGG
```

Rap1 binding site

Figure S1. Sequence of telomeric DNA examined in this study

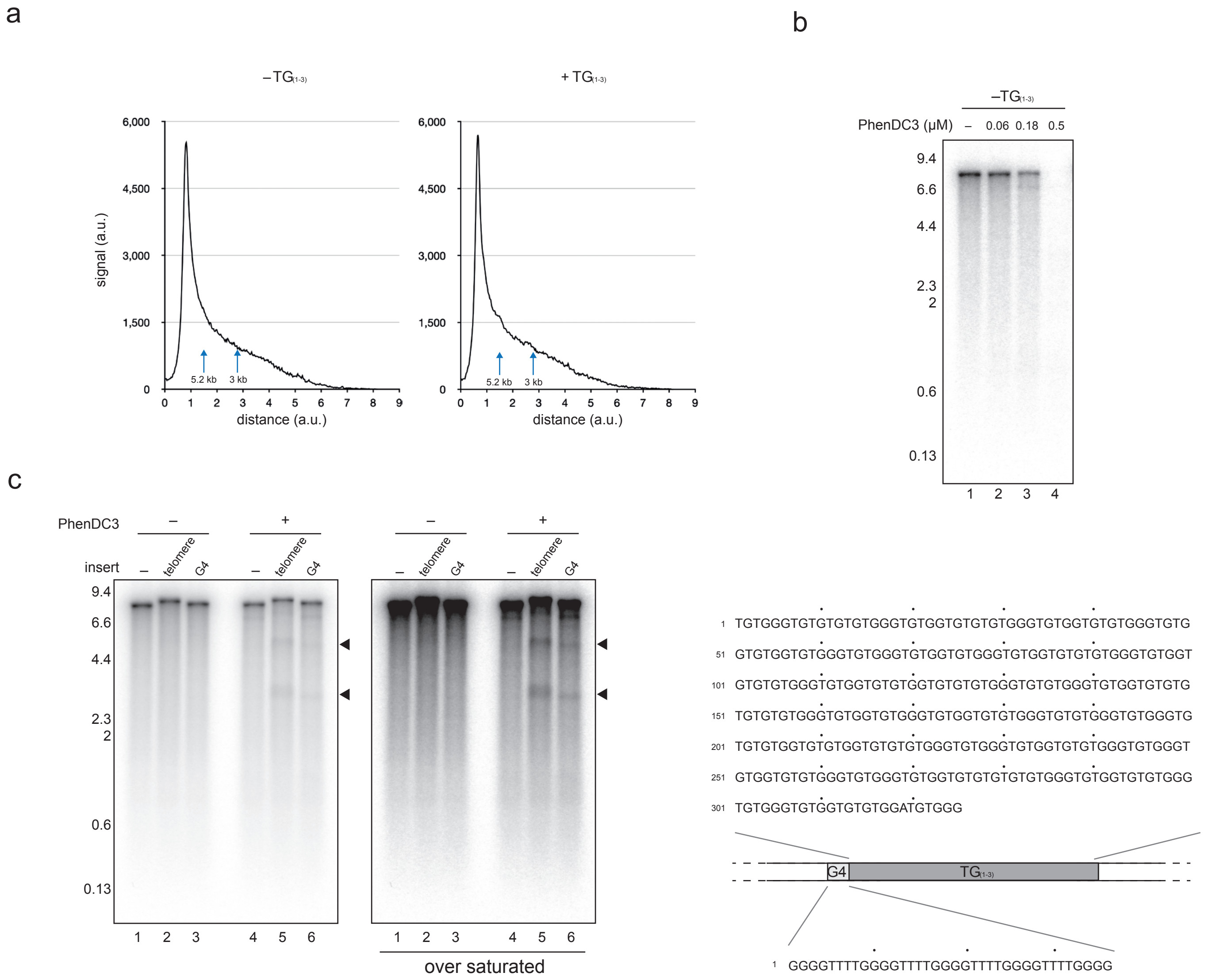

Figure S2. Phen-DC3 can induce sequence-specific defects in lagging strand replication.

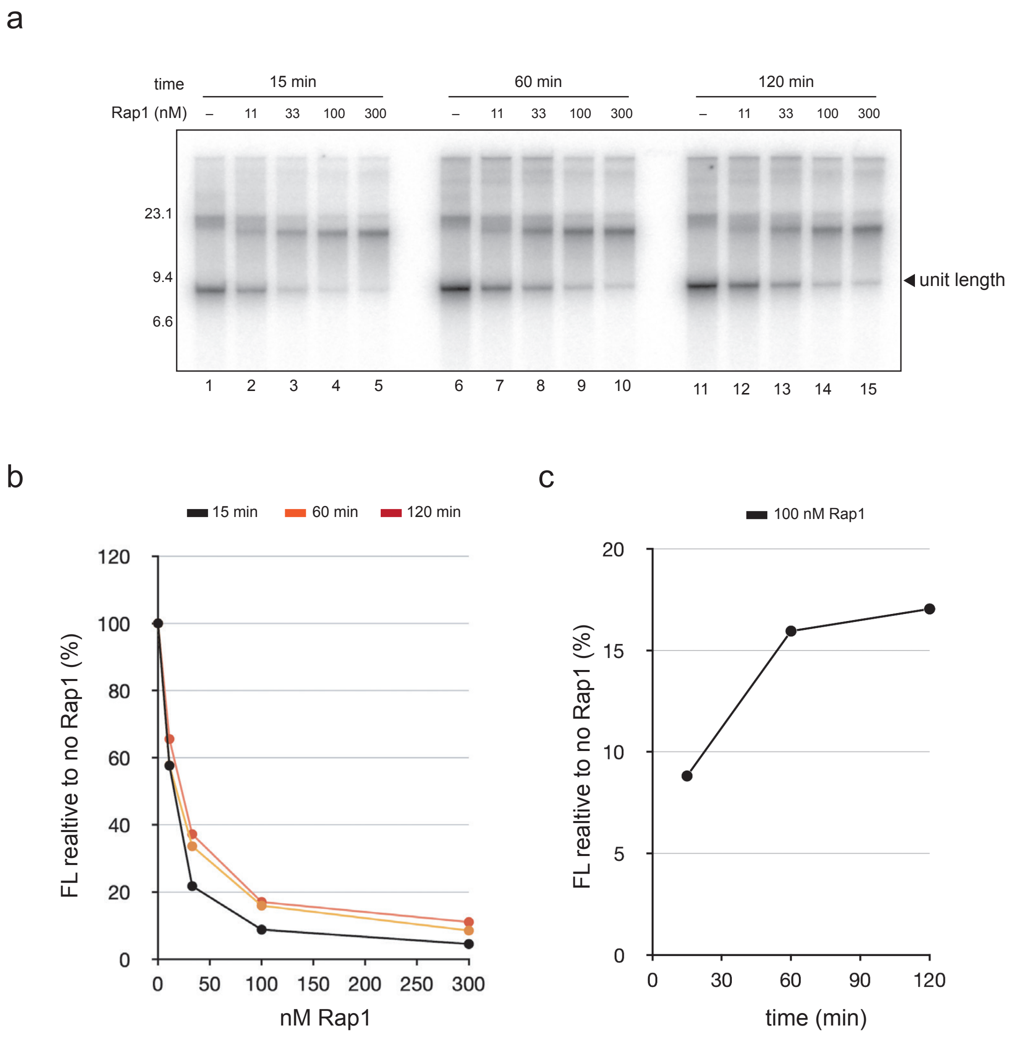

Figure S3. The Rap1 block to DNA replication is largely stable over time

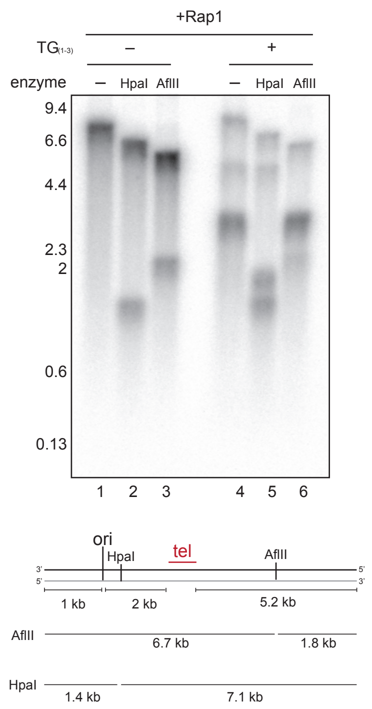

Figure S4. Restriction enzyme analysis of replication products in the presence of Rap1, Fen1 and Cdc9

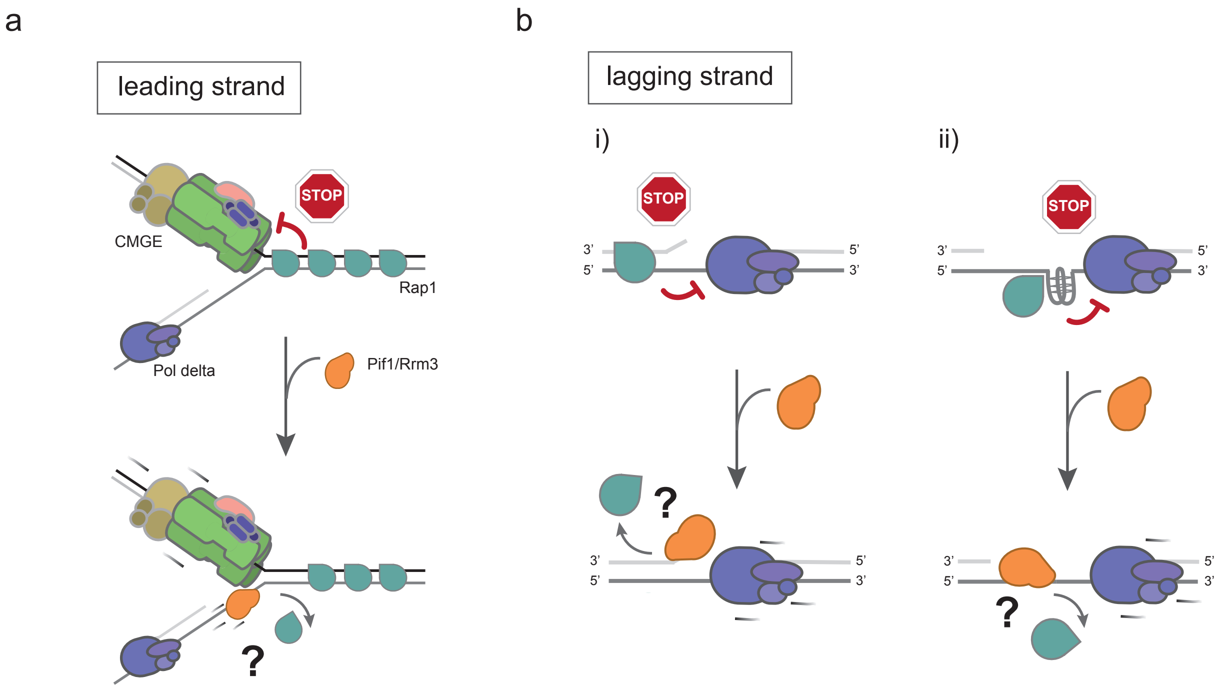

Figure S5. Working models of the impact of Rap1 and Pif1 on replication of telomeric DNA
